# Controlling of Applied Force and Cornea Displacement Estimation in Robotic Corneal Surgery With a Gripper Surgical Instrument

**DOI:** 10.1002/rcs.70038

**Published:** 2025-01-06

**Authors:** Ali Soltani Sharif Abadi, Andrew Ordys, Barbara Pierscionek

**Affiliations:** ^1^ Institute of Electronic Systems Faculty of Electronics and Information Technology Warsaw University of Technology Warsaw Poland; ^2^ The Institute of Automatic Control and Robotics Faculty of Mechatronics Warsaw University of Technology Warsaw Poland; ^3^ Faculty of Health, Education, Medicine and Social Care Medical Technology Research Centre The Institute of Excellence in Robotic Surgery Anglia Ruskin University Chelmsford UK

**Keywords:** fixed‐time control, force estimation, gripper, human eye displacement, robotic eye surgery

## Abstract

**Background:**

The human eye consists of highly sensitive, hydrated, and relatively thin tissues, making precise control and accurate force estimation crucial in robotic eye surgery. This paper introduces a novel control method and state observer designed for a gripper surgical instrument used on the external ocular surface during robotic eye surgery.

**Methods:**

A novel state observer, operating in tandem with the controller, estimates the applied force. The proposed control approach, termed the Fixed‐time Observer‐based Sliding Mode Control (FOSMC), estimates the applied force by determining the gripper states and uses an eye model to calculate its displacement.

**Results:**

The performance of the proposed control method was compared with two other finite‐time and asymptotic techniques across two scenarios. The results demonstrated excellent performance using the proposed method.

**Conclusions:**

The FOSMC control technique effectively estimates the applied force during robotic eye surgery, making it a reliable solution for controlling the gripper surgical instrument.

## 1 Introduction

Robotic eye surgery has a number of practical applications. Robotic systems have the potential to improve the quality of eye surgery compared with routine eye surgery [1]. However, one of the biggest challenges of eye surgery is accuracy [2, 3]. Requirements for accuracy vary depending on the type of eye surgery and this determines the feasibility of utilising robotic surgery [1, 4, 5, 6].

Da Vinci is one of the most popular surgical systems that has also been used for eye surgery. The Da Vinci surgical system consists of three or four manipulator arms that can undertake various surgeries. Two versions of the Da Vinci system, Si [7] and Xi [8], have been developed. Surgical instruments are fitted and conducted to the manipulator arms and are controlled by the Da Vinci's controller. Kudryavtsev et al. [9] developed a vision‐based controller for continuum robot architecture. The controlled robotic structure is based on a three‐tube Concentric Tube Robot (CTR), an emerging paradigm for designing accurate, miniaturised, and flexible endoscopic robots. The authors implemented an eye‐in‐hand visual serving scheme utilising a millimetre‐sized camera embedded at the endoscopic tip. The results demonstrated satisfactory performance in three degrees of freedom positioning and path‐following tasks, incorporating adaptive gain control [9]. Ghoul et al. [10] proposed a dynamic model specifically designed for a cable‐driven continuum robot, addressing key challenges in surgical continuum robotics. The model accounts for the kinematic and dynamic constraints of different robot segments, providing the foundation for a robust and optimised control strategy. This approach facilitates real‐time computation of joint torques based on feedback, ensuring precise and stable task execution even in the presence of external perturbations [10].

A standard surgical instrument used in some types of eye surgeries, such as pterygium and extra‐ocular muscle surgeries, as well as corneal transplantations and lacerations, is the gripper [11, 12, 13]. During the surgery, the gripper has contact with and applies a force to the human eye [14]. The applied force, if too high, can injure the eye, so it must be appropriately controlled. The estimation of the applied force is also helpful in controlling it. Estimations of the applied force of the surgical instrument during different robotic surgeries have been reported [15, 16, 17, 18].

Methods for estimating and controlling the scleral force in robotic eye surgery have been developed [19, 20]. The Adaptive Norm Control (ANC) method was used to estimate the scleral force during robotic retinal surgery [19]. The experimental results of this study showed that the ANC method successfully estimates the scleral force in retinal surgery [19, 20]. In addition to the scleral force, some studies have focused on the external force during robotic surgery [21, 22].

The estimation of the applied force has some advantages, such as reducing the number of sensors, weight of the surgical instrument and construction and maintenance costs of the surgical instrument, and improving the accuracy and quality of the surgery [15, 17, 21, 23]. No estimations of applied force in robotic eye surgery have been found in the literature.

Studies show that the human eye can be modelled as a spring‐damper dynamic model [14, 24, 25]. This model creates a relationship between the applied force and displacement of the human cornea. The data from a state observer can be used to design a suitable force estimator [26, 27]. A state observer estimates the system states, and the estimation can be used for reducing the number of sensors and removing the velocity sensors, thereby more accurately controlling the system. State observers have been designed using methods such as Sliding Mode Finite‐time [28], Fixed‐time Stability [29, 30], command filtered [31], and adaptive fuzzy [32, 33].

This paper describes a robotic gripper for use in ocular surface/corneal surgery. In such surgery, critical features such as stability, precision, and safety‐achieved through a force estimator must be guaranteed by the controller. Hence, an observer‐based sliding mode controller is proposed as a suitable choice. Additionally, a method based on fixed‐time observer‐based sliding mode control provides the fast response essential for this type of surgery. A force estimator, designed using a fixed‐time state observer, will estimate the applied force, with the observer, estimator, and controller operating simultaneously. The main contributions of this paper are as follows:

- Development of a novel control algorithm to track the desired trajectory in the human eye,
- Estimation of the applied force using a fixed‐time state observer,
- Calculation of human corneal displacement during robotic eye surgery,
- Design of a chattering‐free, fixed‐time controller,
- Integration of the state observer, force estimator, and controller to operate concurrently.

## 2 Materials and Methods

### 2.1 Definitions and Lemmas

Definition 1 A sig(∁) is defined by the signum function as sig(∁)=|∁|sign(∁) where sign(∁) is the typical signum function and ∁∈R. The following relations are true [26, 34]:

(1) $$\left\{\begin{array}{c} \begin{array}{c} \text{sign}\left(∁\right)\times \text{sign}\left(∁\right)=1;∁\neq 0\\ \text{si}g^{\iota }\left(∁\right)={\left|∁\right|}^{\iota }\text{sign}\left(∁\right)\; \end{array}\\ ∁\times \text{sign}\left(∁\right)=\left|∁\right|\;\\ \begin{array}{c} ∁\times \text{si}g^{\iota }\left(∁\right)={\left|∁\right|}^{\iota +1}\;\\ \begin{array}{c} \frac{d\left|u\right|}{dt}=\dot{u}\times \text{sign}\left(u\right);\dot{u}=\frac{du}{dt}\;\\ \left|∁\times \text{sign}\left(\varsigma \right)\right|\leq \left|∁\right|\; \end{array} \end{array} \end{array}\right.$$

where the u is a differentiable function and ∁,ς,ι are real numbers as ∁,ς,ι∈R.

Definition 2 Consider the nonlinear system as follows:

(2) $$\dot{x}=f\left(t,x\right);\;x\left(0\right)=x_{0}$$

where $x\in R^{n}$ is the state vector and f(t,x) is a nonlinear function with the state's initial condition $x_{0}$. The origin of the nonlinear system is finite‐time global stable if it is global asymptotical stable and every solution of x(t) of the system reaches the equilibria at a finite‐time, that is $\lim_{t\to T}x=0$ and x=0 for T≤t. The settling time T may depend on the system's initial conditions and control parameters [35, 36].

Definition 3 The origin of the nonlinear system of (2) is fixed‐time global stable, if it is finite‐time global stable, and the settling time T is bounded independently of initial conditions, that is $\exists T>0:T\leq T_{\max}$ [35, 36].

Lemma 1 Assume four real numbers as $\rho_{1},\rho_{3}>0$ and $0<\rho_{2}<1,\rho_{4}>1$, and a Lyapunov candidate function $V:R^{n}\to R^{\geq 0}$ such that; V(x)=0 for x=0. If any solution x(t) of the nonlinear system satisfies the inequality $\dot{V}\leq -\rho_{1}V^{\rho_{2}}-\rho_{3}V^{\rho_{4}}$. Then, it can be proved that the origin of this system has a fixed‐time global stability. Also, the settling time function is $T\leq \frac{1}{\rho_{1}\left(1-\rho_{2}\right)}+\frac{1}{\rho_{3}\left(\rho_{4}-1\right)}$ [36].

Lemma 2 Consider scalar numbers $a_{1},a_{2},\text{…},a_{n}\geq 0$, 0<b≤1 and c>1, then, it can be written [37, 38, 39]:

(3) $$\left\{\begin{array}{c} \sum_{i=1}^{n}a_{i}^{b}\geq {\left(\sum_{i=1}^{n}a_{i}\right)}^{b}\;\\ \sum_{i=1}^{n}a_{i}^{c}\geq n^{1-c}{\left(\sum_{i=1}^{n}a_{i}\right)}^{c} \end{array}\right.$$

Lemma 3 Consider a scalar nonlinear system as follows [26, 40]:

(4) $$\dot{y}=-\alpha y^{p}-\beta y^{m};\;y\left(0\right)=y_{0}$$

where α,β>0,p>0,0<n<1,1<m<2. Then the equilibrium of (6) has a fixed‐time global stability with the settling time $T\leq \frac{1}{\alpha }\frac{1}{1-p}+\frac{1}{\beta }\frac{1}{m-1}$.

### 2.2 Problem Statement

The applied force can potentially cause the human eye to move, which is contraindicated in eye surgery. A dynamic model of the human eye, which includes a relation between the applied force to the eye and displacement of the cornea and has been previously presented [24, 25, 41] is as follows:

(5) $$F=m\ddot{x}\left(t\right)+c\dot{x}\left(t\right)+kx\left(t\right)$$

where F is the applied force to the eye, x is the displacement of the cornea, m is the eye mass, c is the damper coefficient, and k is the spring of the eye. This model was calculated by experimental tests on the human eye. The parameters were determined based on experimental tests in which an air jet applied pressure to the human cornea. These tests provided the empirical data needed to calculate the values of the model parameters, ensuring that they reflect the biomechanical properties of the human eye under the tested conditions [24, 25, 41].

**FIGURE 1:**
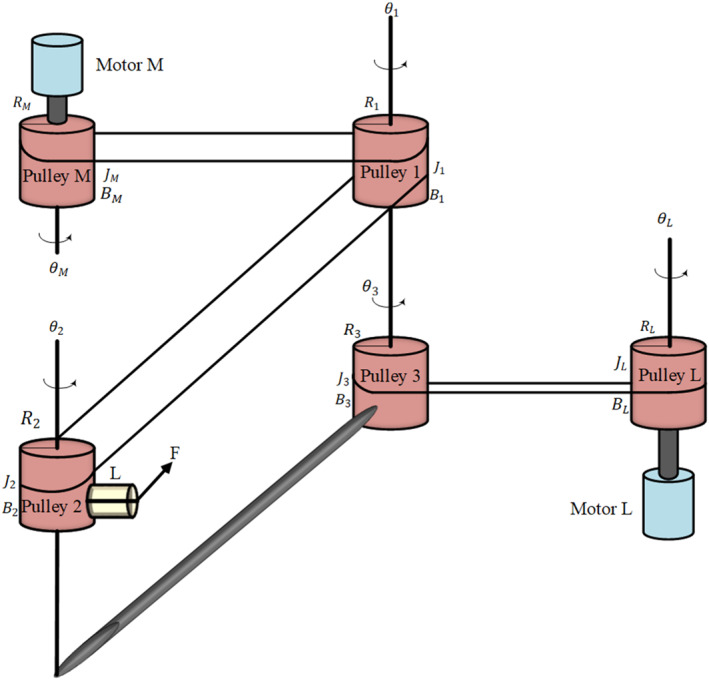
The 2 DOF tendon‐driven gripper [42].

A dynamic model of the gripper, which is a 2‐DOF tendon‐driven system, has been described previously [42] and is shown in Figure 1.

The model consists of three pulleys, and a driving pulley for the end‐effector (M) and pitching link (L). The model of the gripper is as follows [42]:

(6) $$\left\{\begin{array}{c} J_{M}{\ddot{\theta }}_{M}+B_{M}{\dot{\theta }}_{M}+R_{M}{\bar{T}}_{M_{1}}\left(R_{M}\theta_{M}-R_{1}\theta_{1}\right)=u_{M}\\ J_{L}{\ddot{\theta }}_{L}+B_{L}{\dot{\theta }}_{L}+R_{L}{\bar{T}}_{L_{3}}\left(R_{L}\theta_{L}-R_{3}\theta_{3}\right)=u_{L}\; \end{array}\right.$$

where $\theta_{M}$ and $\theta_{L}$ are the angles of the pulleys, $u_{M}$ and $u_{L}$ are the input torques from the motors, $J_{M}$ and $J_{L}$ are the inertia of the pulleys, $B_{M}$ and $B_{L}$ are the damping values of the pulley. $R_{M}$ and $R_{L}$ are the radius of the pulleys. ${\bar{T}}_{M_{1}}$ and ${\bar{T}}_{L_{3}}$ are the functions of the differences in tensions, which acts to transmit the force to the connected pulleys. The dynamic model of the pulleys is presented as follows:

(7) $$\left\{\begin{array}{l} J_{1}{\ddot{\theta }}_{1}+B_{1}{\dot{\theta }}_{1}+R_{1}{\bar{T}}_{12}\left(R_{1}\theta_{1}-R_{1}\theta_{3}-R_{2}\theta_{2}\right)=R_{1}{\bar{T}}_{M_{1}}\left(R_{M}\theta_{M}-R_{1}\theta_{1}\right)\\ J_{2}{\ddot{\theta }}_{2}+B_{2}{\dot{\theta }}_{2}+LF=R_{2}{\bar{T}}_{12}\left(R_{1}\theta_{1}-R_{1}\theta_{3}-R_{2}\theta_{2}\right)\;\\ J_{3}{\ddot{\theta }}_{3}+B_{3}{\dot{\theta }}_{3}+R_{1}{\bar{T}}_{12}\left(R_{1}\theta_{1}-R_{1}\theta_{3}-R_{2}\theta_{2}\right)=R_{3}{\bar{T}}_{L_{3}}\left(R_{L}\theta_{L}-R_{3}\theta_{3}\right)\; \end{array}\right.$$

where $\theta_{j}$ for j=(1,2,3) are the angles of the pulleys, $J_{j}$ are the inertia of the pulleys, $B_{j}$ are the damping values of the pulley, and $R_{j}$ are the radius of the pulleys. ${\bar{T}}_{12}$ is the function of the difference in tension. Also, L is the length of the gripper, and F is the applied force by the gripper.

Note 1 The angles of pulleys 1 and 2 are equal as $\theta_{1}=\theta_{2}$.

This study aims to estimate the applied force of the gripper in eye surgery on the corneal surface. A state observer will be employed to design the force estimator. After estimating the applied force, the displacement of the ocular surface is estimated. Also, the gripper should track the desired trajectory generated by the surgical robot. For this goal, the model of the gripper should become the state space. The model of the gripper can be written as follows:

(8) $$\left\{\begin{array}{l} {\ddot{\theta }}_{M}=\left(\frac{1}{J_{M}}\right)\left(u_{M}-B_{M}{\dot{\theta }}_{M}-R_{M}{\bar{T}}_{M_{1}}\left(R_{M}\theta_{M}-R_{1}\theta_{1}\right)\right)\\ {\ddot{\theta }}_{L}=\left(\frac{1}{J_{L}}\right)\left(u_{L}-B_{L}{\dot{\theta }}_{L}-R_{L}{\bar{T}}_{L_{3}}\left(R_{L}\theta_{L}-R_{3}\theta_{3}\right)\right)\; \end{array}\right.$$

Considering the following states:

(9) $$\left\{\begin{array}{l} \begin{array}{l} \theta_{M}=x_{1}^{M}\\ {\dot{\theta }}_{M}=x_{2}^{M} \end{array}\\ \begin{array}{c} \theta_{L}=x_{1}^{L}\\ {\dot{\theta }}_{L}=x_{2}^{L} \end{array}\; \end{array}\right.$$

The state space of the gripper model will be:

(10) $$\left\{\begin{array}{l} \begin{array}{l} {\dot{x}}_{1}^{M}=x_{2}^{M}\;\\ {\dot{x}}_{2}^{M}=\left(\frac{1}{J_{M}}\right)\left(u_{M}-B_{M}x_{2}^{M}-R_{M}{\bar{T}}_{M_{1}}\left(R_{M}x_{1}^{M}-R_{1}\theta_{1}\right)\right) \end{array}\\ \begin{array}{l} {\dot{x}}_{1}^{L}=x_{2}^{L}\;\\ {\dot{x}}_{2}^{L}=\left(\frac{1}{J_{L}}\right)\left(u_{L}-B_{L}x_{2}^{L}-R_{L}{\bar{T}}_{L_{3}}\left(R_{L}x_{1}^{L}-R_{3}\theta_{3}\right)\right) \end{array}\; \end{array}\right.$$

Also, the dynamic model of the pulleys can be written as the following equations:

(11) $$\left\{\begin{array}{l} J_{1}{\ddot{\theta }}_{1}+B_{1}{\dot{\theta }}_{1}+R_{1}{\bar{T}}_{12}\left(R_{1}\theta_{1}-R_{1}\theta_{3}-R_{2}\theta_{2}\right)=R_{1}{\bar{T}}_{M_{1}}\left(R_{M}x_{1}^{M}-R_{1}\theta_{1}\right)\\ J_{2}{\ddot{\theta }}_{2}+B_{2}{\dot{\theta }}_{2}+LF=R_{2}{\bar{T}}_{12}\left(R_{1}\theta_{1}-R_{1}\theta_{3}-R_{2}\theta_{2}\right)\;\\ J_{3}{\ddot{\theta }}_{3}+B_{3}{\dot{\theta }}_{3}+R_{1}{\bar{T}}_{12}\left(R_{1}\theta_{1}-R_{1}\theta_{3}-R_{2}\theta_{2}\right)=R_{3}{\bar{T}}_{L_{3}}\left(R_{L}x_{1}^{L}-R_{3}\theta_{3}\right)\; \end{array}\right.$$

and

(12) $$\left\{\begin{array}{l} {\ddot{\theta }}_{1}=\left(\frac{1}{J_{1}}\right)\left(R_{1}{\bar{T}}_{M_{1}}\left(R_{M}x_{1}^{M}-R_{1}\theta_{1}\right)-B_{1}{\dot{\theta }}_{1}-R_{1}{\bar{T}}_{12}\left(R_{1}\theta_{1}-R_{1}\theta_{3}-R_{2}\theta_{2}\right)\right)\\ \begin{array}{l} {\ddot{\theta }}_{2}=\left(\frac{1}{J_{2}}\right)\left(R_{2}{\bar{T}}_{12}\left(R_{1}\theta_{1}-R_{1}\theta_{3}-R_{2}\theta_{2}\right)-B_{2}{\dot{\theta }}_{2}-LF\right)\;\\ {\ddot{\theta }}_{3}=\left(\frac{1}{J_{3}}\right)\left(R_{3}{\bar{T}}_{L_{3}}\left(R_{L}x_{1}^{L}-R_{3}\theta_{3}\right)-B_{3}{\dot{\theta }}_{3}-R_{1}{\bar{T}}_{12}\left(R_{1}\theta_{1}-R_{1}\theta_{3}-R_{2}\theta_{2}\right)\right)\; \end{array} \end{array}\right.$$

Considering the following states of the pulleys:

(13) $$\left\{\begin{array}{c} Z_{1}^{1}=\theta_{1}\\ \begin{array}{c} Z_{2}^{1}={\dot{\theta }}_{1}\\ Z_{1}^{2}=\theta_{2} \end{array}\\ \begin{array}{c} Z_{2}^{2}={\dot{\theta }}_{2}\\ Z_{1}^{3}=\theta_{3} \end{array}\\ \begin{array}{c} Z_{2}^{3}={\dot{\theta }}_{3} \end{array} \end{array}\right.\;\text{or}\;\left\{\begin{array}{c} Z_{1}^{j}=\theta_{j}\\ Z_{2}^{j}={\dot{\theta }}_{j} \end{array}\right.\;;\;j=\left(1,2,3\right)$$

The state space of the pulleys will be:

(14) $$\left\{\begin{array}{l} {\dot{Z}}_{1}^{1}=Z_{2}^{1}\;\\ \begin{array}{l} \begin{array}{l} {\dot{Z}}_{2}^{1}=\left(\frac{1}{J_{1}}\right)\left(R_{1}{\bar{T}}_{M_{1}}\left(R_{M}x_{1}^{M}-R_{1}Z_{1}^{1}\right)-B_{1}Z_{2}^{1}-R_{1}{\bar{T}}_{12}\left(R_{1}Z_{1}^{1}-R_{1}Z_{1}^{3}-R_{2}Z_{1}^{2}\right)\right)\\ {\dot{Z}}_{1}^{2}=Z_{2}^{2}\; \end{array}\\ \begin{array}{l} {\dot{Z}}_{2}^{2}=\left(\frac{1}{J_{2}}\right)\left(R_{2}{\bar{T}}_{12}\left(R_{1}Z_{1}^{1}-R_{1}Z_{1}^{3}-R_{2}Z_{1}^{2}\right)-B_{2}Z_{2}^{2}-LF\right)\;\\ {\dot{Z}}_{1}^{3}=Z_{2}^{3}\; \end{array}\\ {\dot{Z}}_{2}^{3}=\left(\frac{1}{J_{3}}\right)\left(R_{3}{\bar{T}}_{L_{3}}\left(R_{L}x_{1}^{L}-R_{3}Z_{1}^{3}\right)-B_{3}Z_{2}^{3}-R_{1}{\bar{T}}_{12}\left(R_{1}Z_{1}^{1}-R_{1}Z_{1}^{3}-R_{2}Z_{1}^{2}\right)\right)\; \end{array} \end{array}\right.$$

The goals of the paper can be written from a mathematical point of view by defining the tracking and estimation errors as follows:

(15) $$\left\{\begin{array}{c} e_{i}^{M}=x_{i}^{M}-x_{i_{d}}^{M}\\ e_{i}^{L}=x_{i}^{L}-x_{i_{d}}^{L}\;\\ {\tilde{x}}_{i}^{M}={\hat{x}}_{i}^{M}-x_{i}^{M}\\ {\tilde{x}}_{i}^{L}={\hat{x}}_{i}^{L}-x_{i}^{L}\;\\ {\tilde{Z}}_{i}^{j}={\hat{Z}}_{i}^{j}-Z_{i}^{j}\; \end{array}\right.$$

where $e_{i}^{M}$ and $e_{i}^{L}$ for i=(1,2) are the tracking errors, as well as ${\tilde{x}}_{i}^{M}$ and ${\tilde{x}}_{i}^{L}$ are the estimation errors. $x_{i_{d}}^{M}$ and $x_{i_{d}}^{L}$ are the desired trajectories which generate by the surgical robot, also, ${\hat{x}}_{i}^{M}$ and ${\hat{x}}_{i}^{L}$ are the estimation of the states. ${\tilde{Z}}_{i}^{j}$ for j=(1,2,3) are the estimation errors of the pulleys and ${\hat{Z}}_{i}^{j}$ are their estimations.

Remark 1 Considering the system's dynamics, the desired trajectories should be as ${\dot{x}}_{1_{d}}^{L}=x_{2_{d}}^{L}$ and ${\dot{x}}_{1_{d}}^{M}=x_{2_{d}}^{M}$.

Assumption 1 In practical applications, we can assume that we know the upper bound of the velocity [26, 43]. In this paper, we can assume that the upper bound of $x_{2}^{L}$ and $x_{2}^{M}$ are available as follows:

(16) $$\left\{\begin{array}{l} \left|x_{2}^{L}\right|\leq \eta_{L}\;\\ \begin{array}{l} \begin{array}{l} \left|x_{2}^{M}\right|\leq \eta_{M}\\ \left|Z_{2}^{1}\right|\leq \eta_{1}\; \end{array}\\ \left|Z_{2}^{2}\right|\leq \eta_{2}\;\\ \left|Z_{2}^{3}\right|\leq \eta_{3}\; \end{array} \end{array}\right.$$

where $\eta_{L}$ and $\eta_{M}$ are two positive constant parameters.

### 2.3 Design of the Force Estimator and Controller

A suitable controller that allows the gripper to track the desired trajectories of the pulleys and for the state observers to estimate the velocity and position of the pulleys is needed. The applied force of the gripper on the human cornea will be estimated by estimating the velocity and position of the pulleys. For this goal, the Sliding Mode Control method is employed. The designed sliding surfaces are as follows:

(17) $$\left\{\begin{array}{l} \begin{array}{l} S_{e_{1}}^{M}={\dot{e}}_{1}^{M}+\phi \left(e_{1}^{M}\right)\;\\ S_{S}^{M}={\dot{S}}_{e_{1}}^{M}+\phi \left(S_{e_{1}}^{M}\right)\; \end{array}\\ \begin{array}{l} S_{e_{1}}^{L}={\dot{e}}_{1}^{L}+\phi \left(e_{1}^{L}\right)\;\\ S_{S}^{L}={\dot{S}}_{e_{1}}^{L}+\phi \left(S_{e_{1}}^{L}\right)\; \end{array} \end{array}\right.$$

where $\phi \left(\varrho \right)=\alpha_{1}^{\varrho }\varrho^{\alpha_{2}^{\varrho }}+\alpha_{3}^{\varrho }\varrho^{\alpha_{4}^{\varrho }}$ for $\varrho =\left(e_{1}^{M},S_{e_{1}}^{M},e_{1}^{L},S_{e_{1}}^{L}\right),$ and $\alpha_{1}^{\varrho },\alpha_{3}^{\varrho }$ are positive control parameters, and other control parameters are as $0<\alpha_{2}^{\varrho }<1$ and $1<\alpha_{4}^{\varrho }<2$.

Remark 2 The designed sliding surface Equation (17) is a type of cascade sliding surface. It means the derivation of the $S_{e_{1}}^{M}$ and $S_{e_{1}}^{L}$ use in $S_{S}^{M}$ and $S_{S}^{L}$ respectively. By applying the derivation of $S_{e_{1}}^{M}$ and $S_{e_{1}}^{L}$ to $S_{S}^{M}$ and $S_{S}^{L}$ can be written:

(18) $$\left\{\begin{array}{c} {\dot{S}}_{S}^{M}={\dot{e}}_{2}^{M}+\dot{\phi }\left(e_{1}^{M}\right)+\phi \left(S_{e_{1}}^{M}\right)\\ {\dot{S}}_{S}^{L}={\dot{e}}_{2}^{L}+\dot{\phi }\left(e_{1}^{L}\right)+\phi \left(S_{e_{1}}^{L}\right)\; \end{array}\right.\;⟹\left\{\begin{array}{l} {\dot{S}}_{S}^{M}=\left(\frac{1}{J_{M}}\right)\left(u_{M}-B_{M}x_{2}^{M}-R_{M}{\bar{T}}_{M_{1}}\left(R_{M}\theta_{M}-R_{1}\theta_{1}\right)\right)-{\dot{x}}_{2_{d}}^{M}\\ +\dot{\phi }\left(e_{1}^{M}\right)+\phi \left(S_{e_{1}}^{M}\right)\\ {\dot{S}}_{S}^{L}=\left(\frac{1}{J_{L}}\right)\left(u_{L}-B_{L}x_{2}^{L}-R_{L}{\bar{T}}_{L_{3}}\left(R_{L}\theta_{L}-R_{3}\theta_{3}\right)\right)-{\dot{x}}_{2_{d}}^{L}\\ +\dot{\phi }\left(e_{1}^{L}\right)+\phi \left(S_{e_{1}}^{L}\right)\; \end{array}\right.$$

The proposed controller is designed as follows:

(19) $$\left\{\begin{array}{l} u_{M}=J_{M}\left(\frac{B_{M}}{J_{M}}{\hat{x}}_{2}^{M}+\frac{R_{M}{\bar{T}}_{M_{1}}}{J_{M}}\left(R_{M}x_{1}^{M}-R_{1}\theta_{1}\right)+{\dot{x}}_{2_{d}}^{M}-\dot{\phi }\left(e_{1}^{M}\right)-\phi \left(S_{e_{1}}^{M}\right)+u_{M}^{t}\right)\\ {\dot{u}}_{M}^{t}=\text{sign}\left(S_{S}^{M}\right)\left(-\left({\left(\frac{B_{M}}{J_{M}}\right)}^{2}+1\right)\left(\left|{\hat{x}}_{2}^{M}+\eta_{M}\right|\right)+u_{M}^{eq}\right)\;\\ u_{M}^{eq}=-\rho_{1}^{M}{\left|S_{S}^{M}\right|}^{\rho_{2}^{M}}-\rho_{3}^{M}{\left|S_{S}^{M}\right|}^{\rho_{4}^{M}}\;\\ u_{L}=J_{L}\left(\frac{B_{L}}{J_{L}}{\hat{x}}_{2}^{L}+\frac{R_{L}{\bar{T}}_{L_{3}}}{J_{L}}\left(R_{L}x_{1}^{L}-R_{3}\theta_{3}\right)+{\dot{x}}_{2_{d}}^{L}-\dot{\phi }\left(e_{1}^{L}\right)-\phi \left(S_{e_{1}}^{L}\right)+u_{L}^{t}\right)\;\\ {\dot{u}}_{L}^{t}=\text{sign}\left(S_{S}^{L}\right)\left(-\left({\left(\frac{B_{L}}{J_{L}}\right)}^{2}+1\right)\left(\left|{\hat{x}}_{2}^{L}+\eta_{L}\right|\right)+u_{L}^{eq}\right)\;\\ u_{L}^{eq}=-\rho_{1}^{L}{\left|S_{S}^{L}\right|}^{\rho_{2}^{L}}-\rho_{3}^{L}{\left|S_{S}^{L}\right|}^{\rho_{4}^{L}}\; \end{array}\right.$$

where $\rho_{p}^{M},\rho_{p}^{L}$ for p=(1,2,3,4) are the control parameters as follows:

(20) $$\left\{\begin{array}{l} \rho_{1}^{M},\rho_{3}^{M},\rho_{1}^{L},\rho_{3}^{L}>0\\ \begin{array}{l} 0<\rho_{2}^{M},\rho_{2}^{L}<1\;\\ \rho_{4}^{M},\rho_{4}^{L}>1\; \end{array} \end{array}\right.$$

For the estimation of the velocity and position of the pulleys, the estimation errors can be written as

(21) $$\left\{\begin{array}{l} \begin{array}{l} {\dot{\tilde{x}}}_{1}^{M}={\dot{\hat{x}}}_{1}^{M}-{\dot{x}}_{1}^{M}={\dot{\hat{x}}}_{1}^{M}-x_{2}^{M}\;\\ {\dot{\tilde{x}}}_{2}^{M}={\dot{\hat{x}}}_{2}^{M}-{\dot{x}}_{2}^{M}={\dot{\hat{x}}}_{2}^{M}-\left(\frac{1}{J_{M}}\right)\left(u_{M}-B_{M}x_{2}^{M}-R_{M}{\bar{T}}_{M_{1}}\left(R_{M}\theta_{M}-R_{1}\theta_{1}\right)\right) \end{array}\\ \begin{array}{l} {\dot{\tilde{x}}}_{1}^{L}={\dot{\hat{x}}}_{1}^{L}-{\dot{x}}_{1}^{L}={\dot{\hat{x}}}_{1}^{L}-x_{2}^{L}\;\\ \begin{array}{c} {\dot{\tilde{x}}}_{2}^{L}={\dot{\hat{x}}}_{2}^{L}-{\dot{x}}_{2}^{L}={\dot{\hat{x}}}_{2}^{L}-\left(\frac{1}{J_{L}}\right)\left(u_{L}-B_{L}x_{2}^{L}-R_{L}{\bar{T}}_{L_{3}}\left(R_{L}\theta_{L}-R_{3}\theta_{3}\right)\right) \end{array}\; \end{array} \end{array}\right.$$

The proposed state observer is designed as follows:

(22) $$\left\{\begin{array}{l} \begin{array}{l} {\dot{\hat{x}}}_{1}^{M}={\hat{x}}_{2}^{M}+{\hat{\chi }}_{M}\;\\ {\hat{\chi }}_{M}=\text{sign}\left({\tilde{x}}_{1}^{M}\right)\left(-\rho_{1}{\left|\left|{\hat{x}}_{2}^{M}\right|+\eta_{M}\right|}^{\rho_{2}}-\rho_{3}{\left|\left|{\hat{x}}_{2}^{M}\right|+\eta_{M}\right|}^{\rho_{4}}-\rho_{1}{\left|{\tilde{x}}_{1}^{M}\right|}^{\rho_{2}}-\rho_{3}{\left|{\tilde{x}}_{1}^{M}\right|}^{\rho_{4}}\right) \end{array}\\ \begin{array}{l} {\dot{\hat{x}}}_{2}^{M}=\left(\frac{1}{J_{M}}\right)\left(u_{M}-B_{M}{\hat{x}}_{2}^{M}-R_{M}{\bar{T}}_{M_{1}}\left(R_{M}x_{1}^{M}-R_{1}\theta_{1}\right)\right) \end{array}\;\\ {\dot{\hat{x}}}_{1}^{L}={\hat{x}}_{2}^{L}+{\hat{\chi }}_{L}\;\\ {\hat{\chi }}_{L}=\text{sign}\left({\tilde{x}}_{1}^{L}\right)\left(-\rho_{1}{\left|\left|{\hat{x}}_{2}^{L}\right|+\eta_{L}\right|}^{\rho_{2}}-\rho_{3}{\left|\left|{\hat{x}}_{2}^{L}\right|+\eta_{L}\right|}^{\rho_{4}}-\rho_{1}{\left|{\tilde{x}}_{1}^{L}\right|}^{\rho_{2}}-\rho_{3}{\left|{\tilde{x}}_{1}^{L}\right|}^{\rho_{4}}\right)\;\\ {\dot{\hat{x}}}_{2}^{L}=\left(\frac{1}{J_{L}}\right)\left(u_{L}-B_{L}{\hat{x}}_{2}^{L}-R_{L}{\bar{T}}_{L_{3}}\left(R_{L}x_{1}^{L}-R_{3}\theta_{3}\right)\right) \end{array}\right.$$

where $\rho_{p}$ are the control parameters as follows:

(23) $$\left\{\begin{array}{l} \rho_{1},\rho_{3}>0\;\\ \begin{array}{l} 0<\rho_{2}<1\\ \rho_{4}>1\; \end{array} \end{array}\right.$$

The estimation error of the pulleys' states can be written as follows

(24) $$\left\{\begin{array}{l} \begin{array}{l} \begin{array}{l} {\dot{\tilde{Z}}}_{1}^{1}={\dot{\hat{Z}}}_{1}^{1}-{\dot{Z}}_{1}^{1}={\dot{\hat{Z}}}_{1}^{1}-Z_{2}^{1}\;\\ {\dot{\tilde{Z}}}_{2}^{1}={\dot{\hat{Z}}}_{2}^{1}-{\dot{Z}}_{2}^{1}={\dot{\hat{Z}}}_{2}^{1}-\left(\frac{1}{J_{1}}\right)\left(R_{1}{\bar{T}}_{M_{1}}\left(R_{M}x_{1}^{M}-R_{1}Z_{1}^{1}\right)-B_{1}Z_{2}^{1}-R_{1}{\bar{T}}_{12}\left(R_{1}Z_{1}^{1}-R_{1}Z_{1}^{3}-R_{2}Z_{1}^{2}\right)\right) \end{array}\\ {\dot{\tilde{Z}}}_{1}^{2}={\dot{\hat{Z}}}_{1}^{2}-{\dot{Z}}_{1}^{2}={\dot{\hat{Z}}}_{1}^{2}-Z_{2}^{2}\;\\ {\dot{\tilde{Z}}}_{2}^{2}={\dot{\hat{Z}}}_{2}^{2}-{\dot{Z}}_{2}^{2}={\dot{\hat{Z}}}_{2}^{2}-\left(\frac{1}{J_{2}}\right)\left(R_{2}{\bar{T}}_{12}\left(R_{1}Z_{1}^{1}-R_{1}Z_{1}^{3}-R_{2}Z_{1}^{2}\right)-B_{2}Z_{2}^{2}-LF\right)\; \end{array}\\ {\dot{\tilde{Z}}}_{1}^{3}={\dot{\hat{Z}}}_{1}^{3}-{\dot{Z}}_{1}^{3}={\dot{\hat{Z}}}_{1}^{3}-Z_{2}^{3}\;\\ {\dot{\tilde{Z}}}_{2}^{3}={\dot{\hat{Z}}}_{2}^{3}-{\dot{Z}}_{2}^{3}={\dot{\hat{Z}}}_{2}^{3}-\left(\frac{1}{J_{3}}\right)\left(R_{3}{\bar{T}}_{L_{3}}\left(R_{L}x_{1}^{L}-R_{3}Z_{1}^{3}\right)-B_{3}Z_{2}^{3}-R_{1}{\bar{T}}_{12}\left(R_{1}Z_{1}^{1}-R_{1}Z_{1}^{3}-R_{2}Z_{1}^{2}\right)\right)\; \end{array}\right.$$

The estimators of the pulleys' states are as follows:

(25) $$\left\{\begin{array}{l} \begin{array}{l} {\dot{\hat{Z}}}_{1}^{1}={\hat{Z}}_{2}^{1}+\lambda_{1}\;\\ \lambda_{1}=\text{sign}\left({\tilde{Z}}_{1}^{1}\right)\left(-\left(\left|{\hat{Z}}_{2}^{1}+\eta_{1}\right|\right)-\rho_{1}^{Z}{\left(\left|{\hat{Z}}_{2}^{1}+\eta_{1}\right|\right)}^{\rho_{2}^{Z}}-\rho_{3}^{Z}{\left(\left|{\hat{Z}}_{2}^{1}+\eta_{1}\right|\right)}^{\rho_{4}^{Z}}-\rho_{1}^{Z}{\left(\left|{\tilde{Z}}_{1}^{1}\right|\right)}^{\rho_{2}^{Z}}-\rho_{3}^{Z}{\left(\left|{\tilde{Z}}_{1}^{1}\right|\right)}^{\rho_{4}^{Z}}\right)\; \end{array}\\ \begin{array}{l} \begin{array}{l} {\dot{\hat{Z}}}_{2}^{1}=\left(\frac{1}{J_{1}}\right)\left(R_{1}{\bar{T}}_{M_{1}}\left(R_{M}x_{1}^{M}-R_{1}Z_{1}^{1}\right)-B_{1}{\hat{Z}}_{2}^{1}-R_{1}{\bar{T}}_{12}\left(R_{1}Z_{1}^{1}-R_{1}Z_{1}^{3}-R_{2}Z_{1}^{2}\right)\right)\;\\ \begin{array}{l} {\dot{\hat{Z}}}_{1}^{2}={\hat{Z}}_{2}^{2}+\lambda_{2}\;\\ \lambda_{2}=\text{sign}\left({\tilde{Z}}_{1}^{2}\right)\left(-\left(\left|{\hat{Z}}_{2}^{2}+\eta_{2}\right|\right)-\rho_{1}^{Z}{\left(\left|{\hat{Z}}_{2}^{2}+\eta_{2}\right|\right)}^{\rho_{2}^{Z}}-\rho_{3}^{Z}{\left(\left|{\hat{Z}}_{2}^{2}+\eta_{2}\right|\right)}^{\rho_{4}^{Z}}-\rho_{1}^{Z}{\left(\left|{\tilde{Z}}_{1}^{2}\right|\right)}^{\rho_{2}^{Z}}-\rho_{3}^{Z}{\left(\left|{\tilde{Z}}_{1}^{2}\right|\right)}^{\rho_{4}^{Z}}\right) \end{array} \end{array}\\ \begin{array}{l} {\dot{\hat{Z}}}_{2}^{2}=\left(\frac{1}{J_{2}}\right)\left(R_{2}{\bar{T}}_{12}\left(R_{1}\theta_{1}-R_{1}\theta_{3}-R_{2}\theta_{2}\right)-B_{2}{\hat{Z}}_{2}^{2}-L\hat{F}\right)\;\\ \begin{array}{l} {\dot{\hat{Z}}}_{1}^{3}={\hat{Z}}_{2}^{3}+\lambda_{3}\;\\ \lambda_{3}=\text{sign}\left({\tilde{Z}}_{1}^{3}\right)\left(-\left(\left|{\hat{Z}}_{2}^{3}+\eta_{3}\right|\right)-\rho_{1}^{Z}{\left(\left|{\hat{Z}}_{2}^{3}+\eta_{3}\right|\right)}^{\rho_{2}^{Z}}-\rho_{3}^{Z}{\left(\left|{\hat{Z}}_{2}^{3}+\eta_{3}\right|\right)}^{\rho_{4}^{Z}}-\rho_{1}^{Z}{\left(\left|{\tilde{Z}}_{1}^{3}\right|\right)}^{\rho_{2}^{Z}}-\rho_{3}^{Z}{\left(\left|{\tilde{Z}}_{1}^{3}\right|\right)}^{\rho_{4}^{Z}}\right) \end{array} \end{array}\\ {\dot{\hat{Z}}}_{2}^{3}=\left(\frac{1}{J_{3}}\right)\left(R_{3}{\bar{T}}_{L_{3}}\left(R_{L}x_{1}^{L}-R_{3}Z_{1}^{3}\right)-B_{3}{\hat{Z}}_{2}^{3}-R_{1}{\bar{T}}_{12}\left(R_{1}Z_{1}^{1}-R_{1}Z_{1}^{3}-R_{2}Z_{1}^{2}\right)\right)\; \end{array} \end{array}\right.$$

where $\rho_{p}^{Z}$ are control parameters that are as follows:

(26) $$\left\{\begin{array}{l} \rho_{1}^{Z},\rho_{3}^{Z}>0\;\\ \begin{array}{l} 0<\rho_{2}^{Z}<1\\ \rho_{4}^{Z}>1\; \end{array} \end{array}\right.$$

The estimator of the applied force is designed as follows:

(27) $$\hat{F}=\left(\frac{1}{L}\right)\left(-J_{2}{\ddot{\hat{\theta }}}_{2}+R_{2}{\bar{T}}_{12}\left(R_{1}\theta_{1}-R_{1}\theta_{3}-R_{2}\theta_{2}\right)-B_{2}{\dot{\hat{\theta }}}_{2}\right)$$

Theorem 1 The system states of Equation (10) track the desired trajectories using the controller defined in Equation (19). The state observer in Equation (22) and the estimator in Equation (25) estimate the system states, as well as the position and velocity of the pulleys, with fixed‐time stability. The upper bound of the settling time is given in Equation (28).

(28) $$T_{s}\leq \max_{\varrho =\left(e_{1}^{M},S_{e_{1}}^{M},e_{1}^{L},S_{e_{1}}^{L}\right)}\left(\frac{1}{\alpha_{1}^{\varrho }\left(1-\alpha_{2}^{\varrho }\right)}+\frac{1}{\alpha_{3}^{\varrho }\left(\alpha_{4}^{\varrho }-1\right)}\right)+\frac{1}{\Lambda_{1}\left(1-\rho_{2}\right)}+\frac{1}{\frac{\Lambda_{2}}{6^{1-\rho_{4}}}\left(\rho_{4}-1\right)}$$

If the sliding surfaces of Equation (17) reach zero at a fixed time, the tracking errors will also reach zero at a fixed time, allowing the system to track the desired trajectories. To prove the theorem, the following Lyapunov function is considered:

(29) $$V=\left|S_{S}^{M}\right|+\left|S_{S}^{L}\right|+\sum_{i=1}^{2}\sum_{j=1}^{3}\left|{\tilde{Z}}_{i}^{j}\right|+\left|{\tilde{x}}_{i}^{M}\right|+\left|{\tilde{x}}_{i}^{L}\right|$$

after the derivation of the Lyapunov function, we have the following:

(30) $$\dot{V}={\dot{S}}_{S}^{M}\text{sign}\left(S_{S}^{M}\right)+{\dot{S}}_{S}^{L}\text{sign}\left(S_{S}^{L}\right)+\sum_{i=1}^{2}\sum_{j=1}^{3}{\dot{\tilde{Z}}}_{i}^{j}\text{sign}\left({\tilde{Z}}_{i}^{j}\right)+{\dot{\tilde{x}}}_{i}^{M}sign\left({\tilde{x}}_{i}^{M}\right)+{\dot{\tilde{x}}}_{i}^{L}\text{sign}\left({\tilde{x}}_{i}^{L}\right)$$

with putting the Equations (18), (21) and (24) and simplification can be written as

(31) $$\dot{V}=\left({\left(\frac{B_{M}}{J_{M}}\right)}^{2}\left(x_{2}^{M}-{\hat{x}}_{2}^{M}\right)+{\dot{u}}_{M}^{t}\right)\text{sign}\left(S_{S}^{M}\right)+\left({\left(\frac{B_{L}}{J_{L}}\right)}^{2}\left(x_{2}^{L}-{\hat{x}}_{2}^{L}\right)+{\dot{u}}_{L}^{t}\right)\text{sign}\left(S_{S}^{L}\right)+\left({\hat{x}}_{2}^{M}-x_{2}^{M}+{\hat{\chi }}_{M}\right)\text{sign}\left({\tilde{x}}_{1}^{M}\right)+\left(\frac{B_{M}}{J_{M}}\left(x_{2}^{M}-{\hat{x}}_{2}^{M}\right)\right)\text{sign}\left({\tilde{x}}_{2}^{M}\right)+\left({\hat{x}}_{2}^{L}-x_{2}^{L}+{\hat{\chi }}_{L}\right)\text{sign}\left({\tilde{x}}_{1}^{L}\right)+\left(\frac{B_{L}}{J_{L}}\left(x_{2}^{L}-{\hat{x}}_{2}^{L}\right)\right)\text{sign}\left({\tilde{x}}_{2}^{L}\right)+\sum_{j=1}^{3}\left({\hat{Z}}_{2}^{j}-Z_{2}^{j}+\lambda_{j}\right)\text{sign}\left({\tilde{Z}}_{1}^{j}\right)+\left(\frac{B_{j}}{J_{j}}\left(Z_{2}^{j}-{\hat{Z}}_{2}^{j}\right)\right)\text{sign}\left({\tilde{Z}}_{2}^{j}\right)$$

then we can write:

(32) $$\dot{V}\leq {\left(\frac{B_{M}}{J_{M}}\right)}^{2}\left(\left|x_{2}^{M}\right|+\left|{\hat{x}}_{2}^{M}\right|\right)+{\dot{u}}_{M}^{t}sign\left(S_{S}^{M}\right)+{\left(\frac{B_{L}}{J_{L}}\right)}^{2}\left(\left|x_{2}^{L}\right|+\left|{\hat{x}}_{2}^{L}\right|\right)+{\dot{u}}_{L}^{t}sign\left(S_{S}^{L}\right)+\left|x_{2}^{M}\right|+\left|{\hat{x}}_{2}^{M}\right|+{\hat{\chi }}_{M}\text{sign}\left({\tilde{x}}_{1}^{M}\right)-\frac{B_{M}}{J_{M}}\left|{\tilde{x}}_{2}^{M}\right|+\left|x_{2}^{L}\right|+\left|{\hat{x}}_{2}^{L}\right|+{\hat{\chi }}_{L}sign\left({\tilde{x}}_{1}^{L}\right)-\frac{B_{L}}{J_{L}}\left|{\tilde{x}}_{2}^{L}\right|+\sum_{j=1}^{3}\left(\left|{\hat{Z}}_{2}^{j}-Z_{2}^{j}\right|+\lambda_{j}\text{sign}\left({\tilde{Z}}_{1}^{j}\right)-\frac{B_{j}}{J_{j}}\left|{\tilde{Z}}_{2}^{j}\right|\right)$$

after simplification:

(33) $$\dot{V}\leq -\rho_{1}^{M}{\left|S_{S}^{M}\right|}^{\rho_{2}^{M}}-\rho_{3}^{M}{\left|S_{S}^{M}\right|}^{\rho_{4}^{M}}-\rho_{1}^{L}{\left|S_{S}^{L}\right|}^{\rho_{2}^{L}}-\rho_{3}^{L}{\left|S_{S}^{L}\right|}^{\rho_{4}^{L}}-\rho_{1}{\left|\left|{\hat{x}}_{2}^{M}\right|+\eta_{M}\right|}^{\rho_{2}}-\rho_{3}{\left|\left|{\hat{x}}_{2}^{M}\right|+\eta_{M}\right|}^{\rho_{4}}-\rho_{1}{\left|{\tilde{x}}_{1}^{M}\right|}^{\rho_{2}}-\rho_{1}{\left|{\tilde{x}}_{1}^{M}\right|}^{\rho_{2}}-\rho_{1}{\left|\left|{\hat{x}}_{2}^{L}\right|+\eta_{L}\right|}^{\rho_{2}}-\rho_{3}{\left|\left|{\hat{x}}_{2}^{L}\right|+\eta_{L}\right|}^{\rho_{4}}-\rho_{1}{\left|{\tilde{x}}_{1}^{L}\right|}^{\rho_{2}}-\rho_{1}{\left|{\tilde{x}}_{1}^{L}\right|}^{\rho_{2}}-\sum_{j=1}^{3}\left(\rho_{1}^{Z}{\left|{\hat{Z}}_{2}^{j}+\eta_{j}\right|}^{\rho_{2}^{Z}}-\rho_{3}^{Z}{\left|{\hat{Z}}_{2}^{j}+\eta_{j}\right|}^{\rho_{4}^{Z}}-\rho_{1}^{Z}{\left|{\tilde{Z}}_{1}^{j}\right|}^{\rho_{2}^{Z}}-\rho_{3}^{Z}{\left|{\tilde{Z}}_{1}^{j}\right|}^{\rho_{4}^{Z}}\right)$$

then:

(34) $$\dot{V}\leq -\rho_{1}^{M}{\left|S_{S}^{M}\right|}^{\rho_{2}^{M}}-\rho_{3}^{M}{\left|S_{S}^{M}\right|}^{\rho_{4}^{M}}-\rho_{1}^{L}{\left|S_{S}^{L}\right|}^{\rho_{2}^{L}}-\rho_{3}^{L}{\left|S_{S}^{L}\right|}^{\rho_{4}^{L}}-\rho_{1}{\left|{\tilde{x}}_{2}^{M}\right|}^{\rho_{2}}-\rho_{3}{\left|{\tilde{x}}_{2}^{M}\right|}^{\rho_{4}}-\rho_{1}{\left|{\tilde{x}}_{1}^{M}\right|}^{\rho_{2}}-\rho_{1}{\left|{\tilde{x}}_{1}^{M}\right|}^{\rho_{2}}-\rho_{1}{\left|{\tilde{x}}_{2}^{L}\right|}^{\rho_{2}}-\rho_{3}{\left|{\tilde{x}}_{2}^{L}\right|}^{\rho_{4}}-\rho_{1}{\left|{\tilde{x}}_{1}^{L}\right|}^{\rho_{2}}-\rho_{1}{\left|{\tilde{x}}_{1}^{L}\right|}^{\rho_{2}}-\sum_{j=1}^{3}\left(\rho_{1}^{Z}{\left|{\tilde{Z}}_{2}^{j}\right|}^{\rho_{2}^{Z}}+\rho_{3}^{Z}{\left|{\tilde{Z}}_{2}^{j}\right|}^{\rho_{4}^{Z}}+\rho_{1}^{Z}{\left|{\tilde{Z}}_{1}^{j}\right|}^{\rho_{2}^{Z}}+\rho_{3}^{Z}{\left|{\tilde{Z}}_{1}^{j}\right|}^{\rho_{4}^{Z}}\right)$$

by defining $\Lambda_{1}=\min\left(\rho_{1}^{M},\rho_{1}^{L},\rho_{1},\rho_{1}^{Z}\right)$ and $\Lambda_{2}=\min\left(\rho_{3}^{M},\rho_{3}^{L},\rho_{3},\rho_{3}^{Z}\right)$, and assuming $\rho_{2}^{Z}=\rho_{2}^{L}=\rho_{2}^{M}=\rho_{2}$ and $\rho_{4}^{Z}=\rho_{4}^{L}=\rho_{4}^{M}=\rho_{4}$:

(35) $$\dot{V}\leq -\Lambda_{1}\left({\left|S_{S}^{M}\right|}^{\rho_{2}}+{\left|S_{S}^{L}\right|}^{\rho_{2}}+{\left|{\tilde{x}}_{2}^{M}\right|}^{\rho_{2}}+{\left|{\tilde{x}}_{1}^{M}\right|}^{\rho_{2}}+{\left|{\tilde{x}}_{2}^{L}\right|}^{\rho_{2}}+{\left|{\tilde{x}}_{1}^{L}\right|}^{\rho_{2}}+\sum_{j=1}^{3}\left({\left|{\tilde{Z}}_{2}^{j}\right|}^{\rho_{2}}++{\left|{\tilde{Z}}_{1}^{j}\right|}^{\rho_{2}}\right)\right)-\Lambda_{2}\left({\left|S_{S}^{M}\right|}^{\rho_{4}}+{\left|S_{S}^{L}\right|}^{\rho_{4}}+{\left|{\tilde{x}}_{2}^{M}\right|}^{\rho_{4}}+{\left|{\tilde{x}}_{1}^{M}\right|}^{\rho_{4}}+{\left|{\tilde{x}}_{2}^{L}\right|}^{\rho_{4}}+{\left|{\tilde{x}}_{1}^{L}\right|}^{\rho_{4}}+\sum_{j=1}^{3}\left({\left|{\tilde{Z}}_{2}^{j}\right|}^{\rho_{4}}+{\left|{\tilde{Z}}_{1}^{j}\right|}^{\rho_{4}}\right)\right)$$

considering Lemma 2:

(36) $$\dot{V}\leq -\Lambda_{1}{\left(\left|S_{S}^{M}\right|+\left|S_{S}^{L}\right|+\left|{\tilde{x}}_{2}^{M}\right|+\left|{\tilde{x}}_{1}^{M}\right|+\left|{\tilde{x}}_{2}^{L}\right|+\left|{\tilde{x}}_{1}^{L}\right|+\sum_{j=1}^{3}\left(\left|{\tilde{Z}}_{2}^{j}\right|+\left|{\tilde{Z}}_{1}^{j}\right|\right)\right)}^{\rho_{2}}-\frac{\Lambda_{2}}{6^{1-\rho_{4}}}{\left(\left|S_{S}^{M}\right|+\left|S_{S}^{L}\right|+\left|{\tilde{x}}_{2}^{M}\right|+\left|{\tilde{x}}_{1}^{M}\right|+\left|{\tilde{x}}_{2}^{L}\right|+\left|{\tilde{x}}_{1}^{L}\right|+\sum_{j=1}^{3}\left(\left|{\tilde{Z}}_{2}^{j}\right|+\left|{\tilde{Z}}_{1}^{j}\right|\right)\right)}^{\rho_{4}}$$

And it is clear that

(37) $$\dot{V}\leq -\Lambda_{1}V^{\rho_{2}}-\frac{\Lambda_{2}}{6^{1-\rho_{4}}}V^{\rho_{4}}$$

The fixed‐time stability of the controller and state observers is proven by considering Lemma 1. It means:

(38) $$\left\{\begin{array}{c} \begin{array}{c} \begin{array}{c} S_{S}^{M}\to 0\\ S_{S}^{L}\to 0\; \end{array}\\ {\tilde{x}}_{i}^{M}\to 0\\ {\tilde{x}}_{i}^{L}\to 0\; \end{array}\\ {\tilde{Z}}_{i}^{j}\to 0\; \end{array}\right.$$

considering Lemma 3, we can write:

(39) $$\left\{\begin{array}{c} \begin{array}{c} \begin{array}{c} \begin{array}{c} S_{e_{1}}^{M}\to 0 \end{array}\;\\ S_{e_{1}}^{L}\to 0\; \end{array}\\ {\hat{x}}_{i}^{M}\to x_{i}^{M}\\ {\hat{x}}_{i}^{L}\to x_{i}^{L}\; \end{array}\\ {\hat{Z}}_{i}^{j}\to Z_{i}^{j}\; \end{array}\right.\;⟹\;\left\{\begin{array}{c} \begin{array}{c} \begin{array}{c} \begin{array}{c} e_{1}^{M}\to 0 \end{array}\;\\ e_{1}^{L}\to 0\; \end{array}\\ {\hat{x}}_{i}^{M}\to x_{i}^{M}\\ {\hat{x}}_{i}^{L}\to x_{i}^{L}\; \end{array}\\ {\hat{Z}}_{i}^{j}\to Z_{i}^{j}\; \end{array}\right.\;⟹\;\left\{\begin{array}{c} \begin{array}{c} \begin{array}{c} x_{i}^{M}\to x_{i_{d}}^{M}\\ x_{i}^{L}\to x_{i_{d}}^{L}\; \end{array}\\ {\hat{x}}_{i}^{M}\to x_{i}^{M}\\ {\hat{x}}_{i}^{L}\to x_{i}^{L}\; \end{array}\\ {\hat{Z}}_{i}^{j}\to Z_{i}^{j}\; \end{array}\right.$$

The fixed‐time stability of Theorem 1 is proven. ∎

Considering Note 1, we can write:

(40) $$\theta_{1}=\theta_{2}⟹{\dot{\theta }}_{1}={\dot{\theta }}_{2}⟹{\ddot{\theta }}_{1}={\ddot{\theta }}_{2}$$

The applied force can be written by using Equation (12) as follows:

(41) $$F=\left(\frac{1}{L}\right)\left(-J_{2}{\ddot{\theta }}_{2}+R_{2}{\bar{T}}_{12}\left(R_{1}\theta_{1}-R_{1}\theta_{3}-R_{2}\theta_{2}\right)-B_{2}{\dot{\theta }}_{2}\right)$$

After proving the fixed‐time stability of the state observers and controller, it is clear that the estimated applied force in Equation (27) will calculate the actual applied force in the fixed time.

## 3 Results

This section presents the simulation results of the proposed controller applied to the gripper. For comparison, two approaches have been selected and modified to ensure compatibility with the proposed method. The comparison methods are chatter‐free, observer‐based, and supported by evidence of finite‐time (terminal) and asymptotic stability. Both comparative methods selected in this study utilise sliding mode‐based techniques. This is for fairness of comparison because of the models used in the design and simulation and the system requirements, as explained in the Introduction. The methods are evaluated under the two scenarios designed to replicate realistic conditions of robotic eye surgery and are as follows:

### 3.1 First Comparative Method

The first method is a validated adaptation of the approach previously published [44]. This method employs a terminal (finite‐time) stabilisation strategy and is referred to as Terminal Observer‐based Sliding Mode Control (TOSMC). The sliding surfaces for the TOSMC method are defined as follows:

(42) $$\left\{\begin{array}{l} \begin{array}{l} S_{e_{1}}^{M}={\dot{e}}_{1}^{M}+\phi \left(e_{1}^{M}\right)\;\\ S_{S}^{M}={\dot{S}}_{e_{1}}^{M}+\phi \left(S_{e_{1}}^{M}\right)\; \end{array}\\ \begin{array}{l} S_{e_{1}}^{L}={\dot{e}}_{1}^{L}+\phi \left(e_{1}^{L}\right)\;\\ S_{S}^{L}={\dot{S}}_{e_{1}}^{L}+\phi \left(S_{e_{1}}^{L}\right)\; \end{array} \end{array}\right.$$

where $\phi \left(\varrho \right)=\alpha_{1}^{\varrho }\varrho^{\alpha_{2}^{\varrho }}$ for $\varrho =\left(e_{1}^{M},S_{e_{1}}^{M},e_{1}^{L},S_{e_{1}}^{L}\right),$ and $0<\alpha_{2}^{\varrho }<1$ and $\alpha_{1}^{\varrho }>0$ are the control parameters.

The controller of the TOSMC method is designed as follows:

(43) $$\left\{\begin{array}{l} u_{M}=J_{M}\left(\frac{B_{M}}{J_{M}}{\hat{x}}_{2}^{M}+\frac{R_{M}{\bar{T}}_{M_{1}}}{J_{M}}\left(R_{M}x_{1}^{M}-R_{1}\theta_{1}\right)+{\dot{x}}_{2_{d}}^{M}-\dot{\phi }\left(e_{1}^{M}\right)-\phi \left(S_{e_{1}}^{M}\right)+u_{M}^{t}\right)\\ {\dot{u}}_{M}^{t}=\text{sign}\left(S_{S}^{M}\right)\left(-\left({\left(\frac{B_{M}}{J_{M}}\right)}^{2}+1\right)\left(\left|{\hat{x}}_{2}^{M}+\eta_{M}\right|\right)+u_{M}^{eq}\right)\;\\ u_{M}^{eq}=-\rho_{1}^{M}{\left|S_{S}^{M}\right|}^{\rho_{2}^{M}}\;\\ u_{L}=J_{L}\left(\frac{B_{L}}{J_{L}}{\hat{x}}_{2}^{L}+\frac{R_{L}{\bar{T}}_{L_{3}}}{J_{L}}\left(R_{L}x_{1}^{L}-R_{3}\theta_{3}\right)+{\dot{x}}_{2_{d}}^{L}-\dot{\phi }\left(e_{1}^{L}\right)-\phi \left(S_{e_{1}}^{L}\right)+u_{L}^{t}\right)\;\\ {\dot{u}}_{L}^{t}=\text{sign}\left(S_{S}^{L}\right)\left(-\left({\left(\frac{B_{L}}{J_{L}}\right)}^{2}+1\right)\left(\left|{\hat{x}}_{2}^{L}+\eta_{L}\right|\right)+u_{L}^{eq}\right)\;\\ u_{L}^{eq}=-\rho_{1}^{L}{\left|S_{S}^{L}\right|}^{\rho_{2}^{L}}\; \end{array}\right.$$

where $\rho_{p}^{M},\rho_{p}^{L}$ for p=(1,2) are the control parameters as follows:

(44) $$\left\{\begin{array}{l} \begin{array}{c} \rho_{1}^{M},\rho_{1}^{L}>0 \end{array}\;\\ 0<\rho_{2}^{M},\rho_{2}^{L}<1 \end{array}\right.$$

The state observer of the TOSMC method is as follows:

(45) $$\left\{\begin{array}{l} {\dot{\hat{x}}}_{1}^{M}={\hat{x}}_{2}^{M}+{\hat{\chi }}_{M}\;\\ {\hat{\chi }}_{M}=\text{sign}\left({\tilde{x}}_{1}^{M}\right)\left(-\rho_{1}{\left|\left|{\hat{x}}_{2}^{M}\right|+\eta_{M}\right|}^{\rho_{2}}-\rho_{1}{\left|{\tilde{x}}_{1}^{M}\right|}^{\rho_{2}}\right)\;\\ {\dot{\hat{x}}}_{2}^{M}=\left(\frac{1}{J_{M}}\right)\left(u_{M}-B_{M}{\hat{x}}_{2}^{M}-R_{M}{\bar{T}}_{M_{1}}\left(R_{M}x_{1}^{M}-R_{1}\theta_{1}\right)\right)\\ {\dot{\hat{x}}}_{1}^{L}={\hat{x}}_{2}^{L}+{\hat{\chi }}_{L}\;\\ {\hat{\chi }}_{L}=\text{sign}\left({\tilde{x}}_{1}^{L}\right)\left(-\rho_{1}{\left|\left|{\hat{x}}_{2}^{L}\right|+\eta_{L}\right|}^{\rho_{2}}-\rho_{1}{\left|{\tilde{x}}_{1}^{L}\right|}^{\rho_{2}}\right)\;\\ {\dot{\hat{x}}}_{2}^{L}=\left(\frac{1}{J_{L}}\right)\left(u_{L}-B_{L}{\hat{x}}_{2}^{L}-R_{L}{\bar{T}}_{L_{3}}\left(R_{L}x_{1}^{L}-R_{3}\theta_{3}\right)\right) \end{array}\right.$$

where $\rho_{p}$ are the control parameters as follows:

(46) $$\left\{\begin{array}{l} \rho_{1}>0\;\\ 0<\rho_{2}<1 \end{array}\right.$$

The estimators of the pulleys' states are as follows:

(47) $$\left\{\begin{array}{l} {\dot{\hat{Z}}}_{1}^{1}={\hat{Z}}_{2}^{1}+\lambda_{1}\;\\ \lambda_{1}=\text{sign}\left({\tilde{Z}}_{1}^{1}\right)\left(-\left(\left|{\hat{Z}}_{2}^{1}+\eta_{1}\right|\right)-\rho_{1}^{Z}{\left(\left|{\hat{Z}}_{2}^{1}+\eta_{1}\right|\right)}^{\rho_{2}^{Z}}-\rho_{1}^{Z}{\left(\left|{\tilde{Z}}_{1}^{1}\right|\right)}^{\rho_{2}^{Z}}\right)\;\\ {\dot{\hat{Z}}}_{2}^{1}=\left(\frac{1}{J_{1}}\right)\left(R_{1}{\bar{T}}_{M_{1}}\left(R_{M}x_{1}^{M}-R_{1}Z_{1}^{1}\right)-B_{1}{\hat{Z}}_{2}^{1}-R_{1}{\bar{T}}_{12}\left(R_{1}Z_{1}^{1}-R_{1}Z_{1}^{3}-R_{2}Z_{1}^{2}\right)\right)\\ {\dot{\hat{Z}}}_{1}^{2}={\hat{Z}}_{2}^{2}+\lambda_{2}\;\\ \lambda_{2}=\text{sign}\left({\tilde{Z}}_{1}^{2}\right)\left(-\left(\left|{\hat{Z}}_{2}^{2}+\eta_{2}\right|\right)-\rho_{1}^{Z}{\left(\left|{\hat{Z}}_{2}^{2}+\eta_{2}\right|\right)}^{\rho_{2}^{Z}}-\rho_{1}^{Z}{\left(\left|{\tilde{Z}}_{1}^{2}\right|\right)}^{\rho_{2}^{Z}}\right)\;\\ {\dot{\hat{Z}}}_{2}^{2}=\left(\frac{1}{J_{2}}\right)\left(R_{2}{\bar{T}}_{12}\left(R_{1}\theta_{1}-R_{1}\theta_{3}-R_{2}\theta_{2}\right)-B_{2}{\hat{Z}}_{2}^{2}-L\hat{F}\right)\;\\ {\dot{\hat{Z}}}_{1}^{3}={\hat{Z}}_{2}^{3}+\lambda_{3}\;\\ \lambda_{3}=\text{sign}\left({\tilde{Z}}_{1}^{3}\right)\left(-\left(\left|{\hat{Z}}_{2}^{3}+\eta_{3}\right|\right)-\rho_{1}^{Z}{\left(\left|{\hat{Z}}_{2}^{3}+\eta_{3}\right|\right)}^{\rho_{2}^{Z}}-\rho_{1}^{Z}{\left(\left|{\tilde{Z}}_{1}^{3}\right|\right)}^{\rho_{2}^{Z}}\right)\;\\ {\dot{\hat{Z}}}_{2}^{3}=\left(\frac{1}{J_{3}}\right)\left(R_{3}{\bar{T}}_{L_{3}}\left(R_{L}x_{1}^{L}-R_{3}Z_{1}^{3}\right)-B_{3}{\hat{Z}}_{2}^{3}-R_{1}{\bar{T}}_{12}\left(R_{1}Z_{1}^{1}-R_{1}Z_{1}^{3}-R_{2}Z_{1}^{2}\right)\right)\; \end{array}\right.$$

where $\rho_{p}^{Z}$ are control parameters that are as follows:

(48) $$\left\{\begin{array}{l} \rho_{1}^{Z}>0\;\\ 0<\rho_{2}^{Z}<1 \end{array}\right.$$

### 3.2 Second Comparative Method

The second method is a validated adaptation of the approach published in [45]. This method employs an asymptotic stabilisation strategy and is referred to as Asymptotically Observer‐based Sliding Mode Control (AOSMC). The sliding surfaces for the AOSMC method are defined as follows:

(49) $$\left\{\begin{array}{l} S_{e_{1}}^{M}={\dot{e}}_{1}^{M}+\phi \left(e_{1}^{M}\right)\;\\ S_{S}^{M}={\dot{S}}_{e_{1}}^{M}+\phi \left(S_{e_{1}}^{M}\right)\;\\ S_{e_{1}}^{L}={\dot{e}}_{1}^{L}+\phi \left(e_{1}^{L}\right)\;\\ S_{S}^{L}={\dot{S}}_{e_{1}}^{L}+\phi \left(S_{e_{1}}^{L}\right)\; \end{array}\right.$$

where $\phi \left(\varrho \right)=\alpha_{1}^{\varrho }\varrho$ for $\varrho =\left(e_{1}^{M},S_{e_{1}}^{M},e_{1}^{L},S_{e_{1}}^{L}\right),$ and $\alpha_{1}^{\varrho }>0$ is the control parameters.

The controller of the AOSMC method is designed as follows:

(50) $$\left\{\begin{array}{l} u_{M}=J_{M}\left(\frac{B_{M}}{J_{M}}{\hat{x}}_{2}^{M}+\frac{R_{M}{\bar{T}}_{M_{1}}}{J_{M}}\left(R_{M}x_{1}^{M}-R_{1}\theta_{1}\right)+{\dot{x}}_{2_{d}}^{M}-\dot{\phi }\left(e_{1}^{M}\right)-\phi \left(S_{e_{1}}^{M}\right)+u_{M}^{t}\right)\\ {\dot{u}}_{M}^{t}=\text{sign}\left(S_{S}^{M}\right)\left(-\left({\left(\frac{B_{M}}{J_{M}}\right)}^{2}+1\right)\left(\left|{\hat{x}}_{2}^{M}+\eta_{M}\right|\right)\right)\;\\ u_{L}=J_{L}\left(\frac{B_{L}}{J_{L}}{\hat{x}}_{2}^{L}+\frac{R_{L}{\bar{T}}_{L_{3}}}{J_{L}}\left(R_{L}x_{1}^{L}-R_{3}\theta_{3}\right)+{\dot{x}}_{2_{d}}^{L}-\dot{\phi }\left(e_{1}^{L}\right)-\phi \left(S_{e_{1}}^{L}\right)+u_{L}^{t}\right)\;\\ {\dot{u}}_{L}^{t}=\text{sign}\left(S_{S}^{L}\right)\left(-\left({\left(\frac{B_{L}}{J_{L}}\right)}^{2}+1\right)\left(\left|{\hat{x}}_{2}^{L}+\eta_{L}\right|\right)\right)\; \end{array}\right.$$

The state observer of the AOSMC method is as follows:

(51) $$\left\{\begin{array}{l} {\dot{\hat{x}}}_{1}^{M}={\hat{x}}_{2}^{M}+{\hat{\chi }}_{M}\;\\ {\hat{\chi }}_{M}=\text{sign}\left({\tilde{x}}_{1}^{M}\right)\left(-\rho_{1}\left|\left|{\hat{x}}_{2}^{M}\right|+\eta_{M}\right|\right)\;\\ {\dot{\hat{x}}}_{2}^{M}=\left(\frac{1}{J_{M}}\right)\left(u_{M}-B_{M}{\hat{x}}_{2}^{M}-R_{M}{\bar{T}}_{M_{1}}\left(R_{M}x_{1}^{M}-R_{1}\theta_{1}\right)\right)\\ {\dot{\hat{x}}}_{1}^{L}={\hat{x}}_{2}^{L}+{\hat{\chi }}_{L}\;\\ {\hat{\chi }}_{L}=\text{sign}\left({\tilde{x}}_{1}^{L}\right)\left(-\rho_{1}\left|\left|{\hat{x}}_{2}^{L}\right|+\eta_{L}\right|\right)\;\\ {\dot{\hat{x}}}_{2}^{L}=\left(\frac{1}{J_{L}}\right)\left(u_{L}-B_{L}{\hat{x}}_{2}^{L}-R_{L}{\bar{T}}_{L_{3}}\left(R_{L}x_{1}^{L}-R_{3}\theta_{3}\right)\right) \end{array}\right.$$

where $\rho_{1}>0$ is a positive control parameter. The estimators of the pulleys' states are as follows:

(52) $$\left\{\begin{array}{l} {\dot{\hat{Z}}}_{1}^{1}={\hat{Z}}_{2}^{1}+\lambda_{1}\;\\ \lambda_{1}=\text{sign}\left({\tilde{Z}}_{1}^{1}\right)\left(-\left(\left|{\hat{Z}}_{2}^{1}+\eta_{1}\right|\right)\right)\;\\ {\dot{\hat{Z}}}_{2}^{1}=\left(\frac{1}{J_{1}}\right)\left(R_{1}{\bar{T}}_{M_{1}}\left(R_{M}x_{1}^{M}-R_{1}Z_{1}^{1}\right)-B_{1}{\hat{Z}}_{2}^{1}-R_{1}{\bar{T}}_{12}\left(R_{1}Z_{1}^{1}-R_{1}Z_{1}^{3}-R_{2}Z_{1}^{2}\right)\right)\\ {\dot{\hat{Z}}}_{1}^{2}={\hat{Z}}_{2}^{2}+\lambda_{2}\;\\ \lambda_{2}=\text{sign}\left({\tilde{Z}}_{1}^{2}\right)\left(-\left(\left|{\hat{Z}}_{2}^{2}+\eta_{2}\right|\right)\right)\;\\ {\dot{\hat{Z}}}_{2}^{2}=\left(\frac{1}{J_{2}}\right)\left(R_{2}{\bar{T}}_{12}\left(R_{1}\theta_{1}-R_{1}\theta_{3}-R_{2}\theta_{2}\right)-B_{2}{\hat{Z}}_{2}^{2}-L\hat{F}\right)\;\\ {\dot{\hat{Z}}}_{1}^{3}={\hat{Z}}_{2}^{3}+\lambda_{3}\;\\ \lambda_{3}=\text{sign}\left({\tilde{Z}}_{1}^{3}\right)\left(-\left(\left|{\hat{Z}}_{2}^{3}+\eta_{3}\right|\right)\right)\;\\ {\dot{\hat{Z}}}_{2}^{3}=\left(\frac{1}{J_{3}}\right)\left(R_{3}{\bar{T}}_{L_{3}}\left(R_{L}x_{1}^{L}-R_{3}Z_{1}^{3}\right)-B_{3}{\hat{Z}}_{2}^{3}-R_{1}{\bar{T}}_{12}\left(R_{1}Z_{1}^{1}-R_{1}Z_{1}^{3}-R_{2}Z_{1}^{2}\right)\right)\; \end{array}\right.$$

### 3.3 First Scenario

The first scenario was evaluated using Simulink/MATLAB software. This scenario aims to demonstrate the gripper surgical instrument ability to track a desired trajectory, to estimate the applied force through system state estimation, and to calculate the displacement of the human eye using the aforementioned eye model. The desired trajectories for this scenario are defined as $x_{1_{d}}^{M}=0.05e^{-t},x_{2_{d}}^{M}=-0.05e^{-t},x_{1_{d}}^{L}=-0.05e^{-t},x_{2_{d}}^{L}=0.05e^{-t}$. This is an example of a trajectory that could be a fragment of a movement of the gripper on the cornea. The parameters of the gripper and human eye model are presented in Table 1. Tables 2, 3, 4 show the tuned control parameters for different methods.

**TABLE 1:** The gripper and human eye model parameters [25, 42].

Parameter	Value	Related equation
m[g]	0.32	Equation (5)
c[Ns/m]	0.13	Equation (5)
k[N/m]	240	Equation (5)
$J_{m},J_{L}\;\left[gm^{2}\right]$	$11.61\times 10^{-4}$	Equation (6)
$J_{1},J_{2},J_{3}\;\left[gm^{2}\right]$	$1.16\times 10^{-4}$	Equation (7)
$B_{m},B_{L}\;\left[Nms/rad\right]$	$2.27\times 10^{-4}$	Equation (6)
$B_{1},B_{2},B_{3}\;\left[Nms/rad\right]$	$2.27\times 10^{-3}$	Equation (7)
$R_{m},R_{L},R_{1},R_{2},R_{3}\;\left[m\right]$	$1\times 10^{-2}$	Equations (6) and (7)
${\bar{T}}_{M_{1}},{\bar{T}}_{L_{3}}\;\left[N/m\right]$	0.35	Equations (6) and (7)
L[m]	$2.8\times 10^{-2}$	Equation (7)

**TABLE 2:** Control parameters used for the proposed FOSMC method.

Parameter	Value	Related equation
$\alpha_{1}^{\varrho },\alpha_{3}^{\varrho }$	20	Equation (17)
$\alpha_{2}^{\varrho }$	101/103	Equation (17)
$\alpha_{4}^{\varrho }$	105/103	Equation (17)
$\rho_{1}^{M},\rho_{1}^{L},\rho_{1},\rho_{1}^{Z}$	2	Equations (19), (22), and (25)
$\rho_{3}^{M},\rho_{3}^{L},\rho_{3},\rho_{3}^{Z}$	1	Equations (19), (22), and (25)
$\rho_{2}^{M},\rho_{2}^{L},\rho_{2},\rho_{2}^{Z}$	101/103	Equations (19), (22), and (25)
$\rho_{4}^{M},\rho_{4}^{L},\rho_{4},\rho_{4}^{Z}$	105/103	Equations (19), (22), and (25)

**TABLE 3:** Control parameters used for the TOSMC method.

Parameter	Value	Related equation
$\alpha_{1}^{\varrho }$	20	Equation (42)
$\alpha_{2}^{\varrho }$	101/103	Equation (42)
$\rho_{1}^{M},\rho_{1}^{L},\rho_{1},\rho_{1}^{Z}$	2	Equations (43), (45), and (47)
$\rho_{2}^{M},\rho_{2}^{L},\rho_{2},\rho_{2}^{Z}$	101/103	Equations (43), (45), and (47)

**TABLE 4:** Control parameters used for the AOSMC method.

Parameter	Value	Related equation
$\alpha_{1}^{\varrho }$	20	Equation (49)
$\rho_{1}^{M},\rho_{1}^{L},\rho_{1},\rho_{1}^{Z}$	2	Equations (50), (51), (52)

All methods were simulated under identical conditions. An ode4 solver with a step size of 0.01 was used for simulations. The initial conditions were set to zero. The simulation results of the first scenario are illustrated in Figure 2, 3, 4, 5, 6, 7, 8, 9, 10, 11, 12, 13, 14, 15, 16, 17.

**FIGURE 2:**
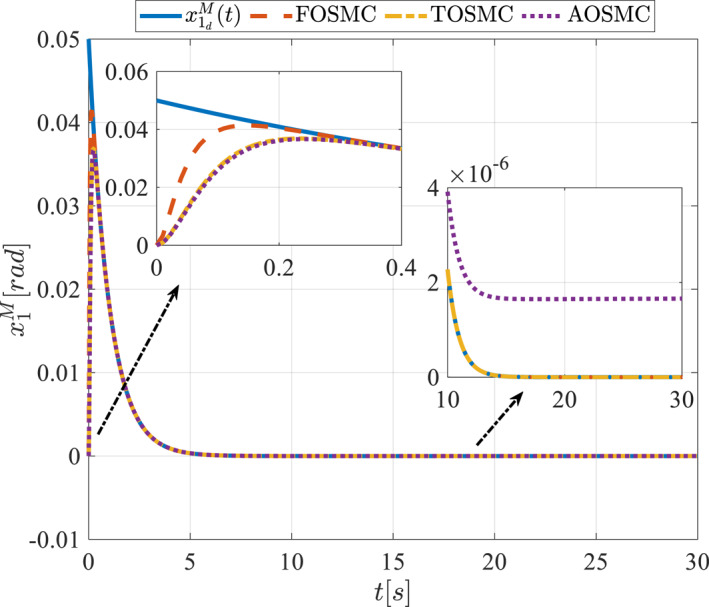
The first state of the driving pulley in the first scenario.

**FIGURE 3:**
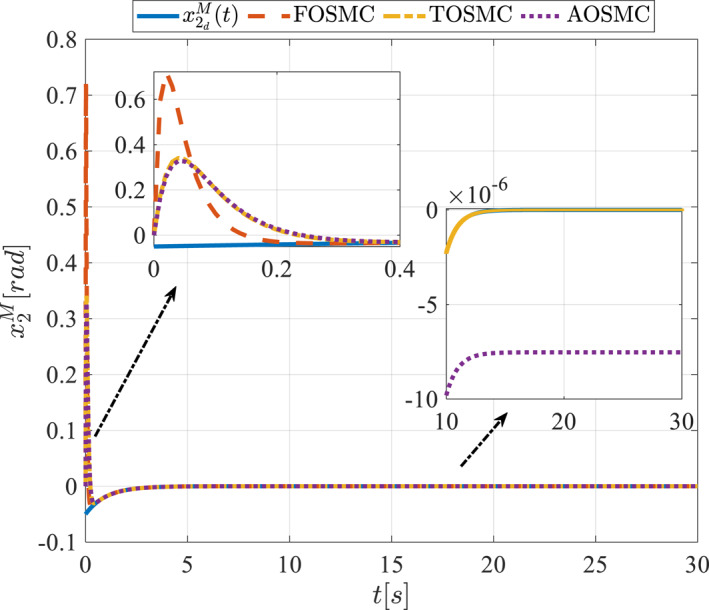
The second state of the driving pulley in the first scenario.

**FIGURE 4:**
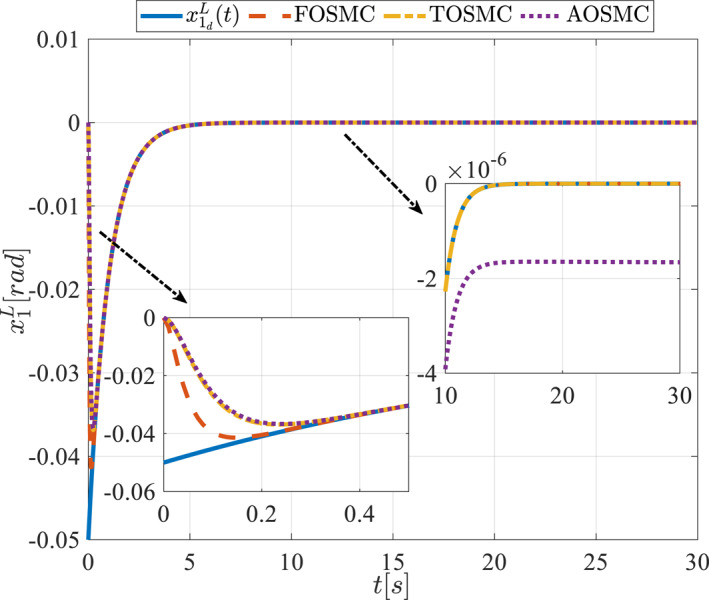
The first state of the pitching link in the first scenario.

**FIGURE 5:**
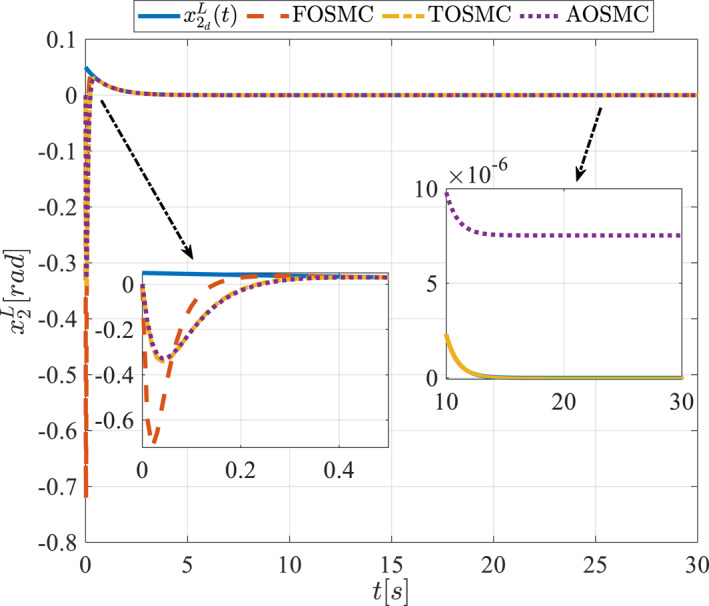
The second state of the pitching link in the first scenario.

**FIGURE 6:**
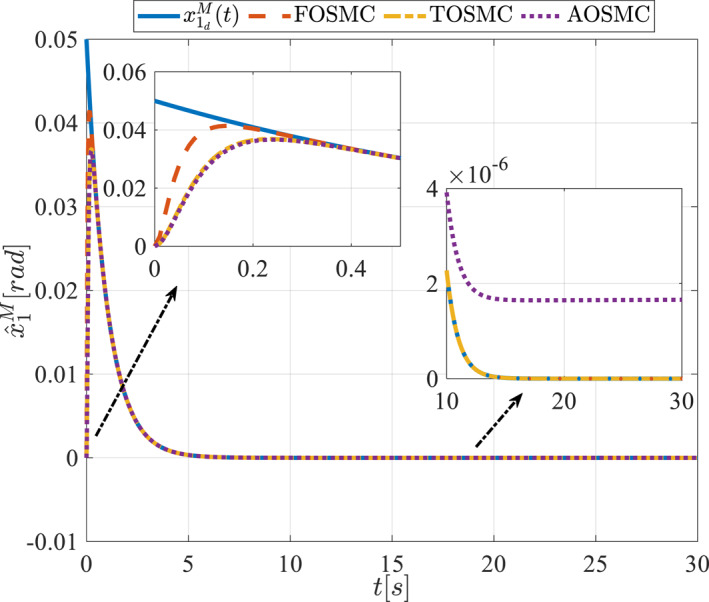
Estimation of the first state of the driving pulley in the first scenario.

**FIGURE 7:**
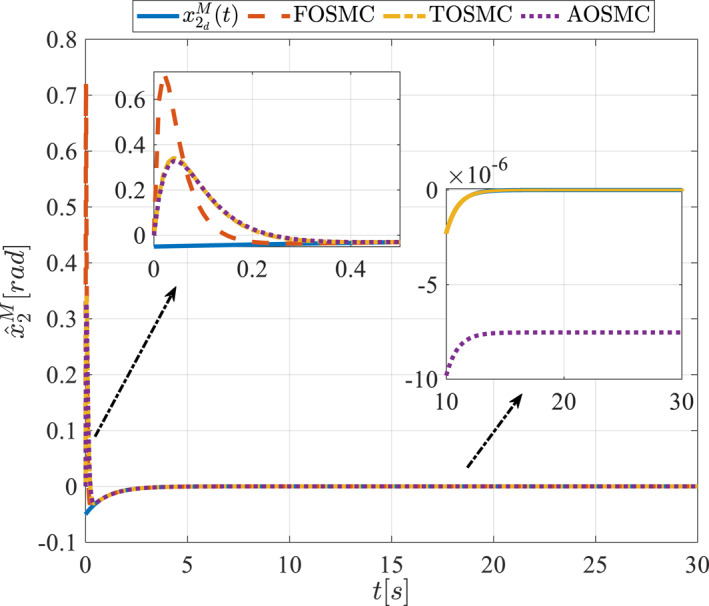
Estimation of the second state of the driving pulley in the first scenario.

**FIGURE 8:**
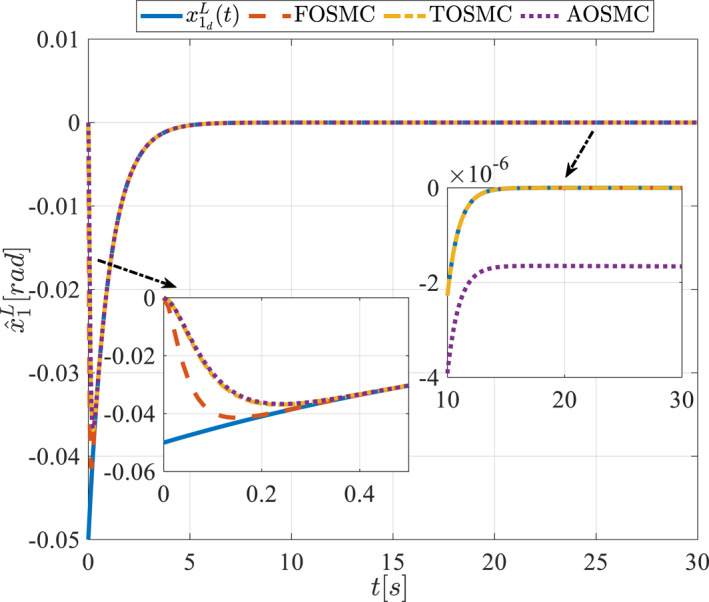
Estimation of the first state of the pitching link in the first scenario.

**FIGURE 9:**
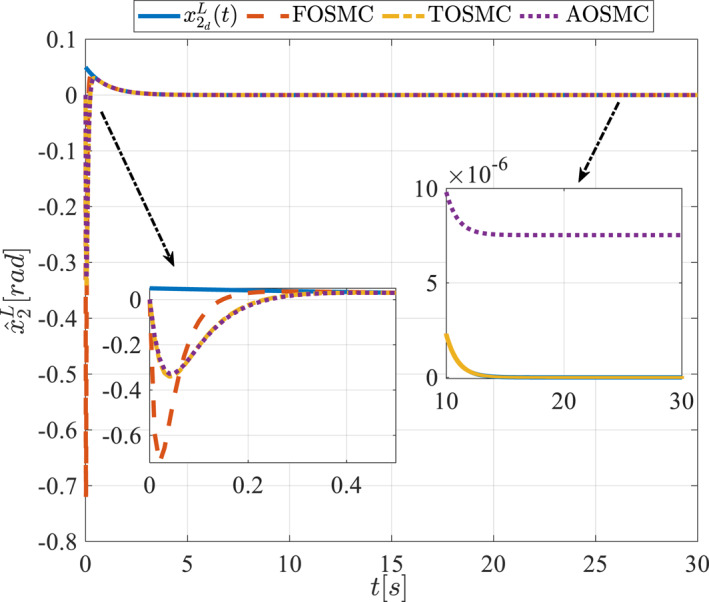
Estimation of the second state of the pitching link in the first scenario.

**FIGURE 10:**
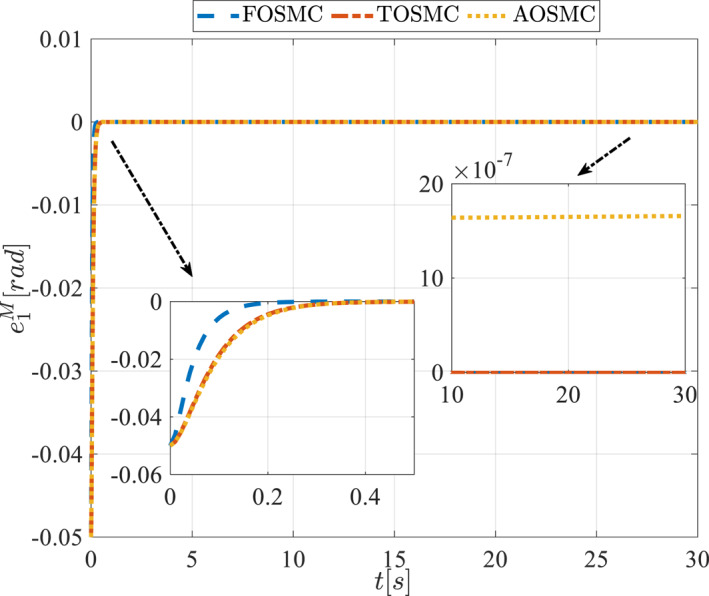
Tracking error of the first state of the driving pulley in the first scenario.

**FIGURE 11:**
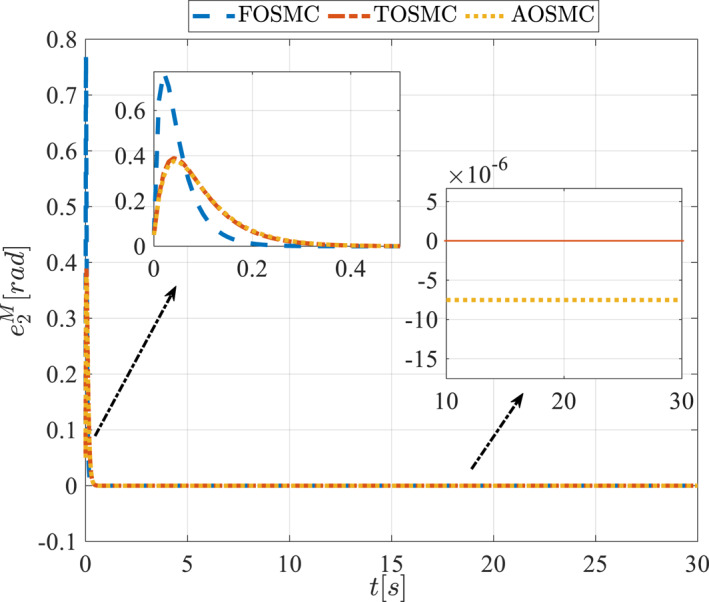
Tracking error of the second state of the driving pulley in the first scenario.

**FIGURE 12:**
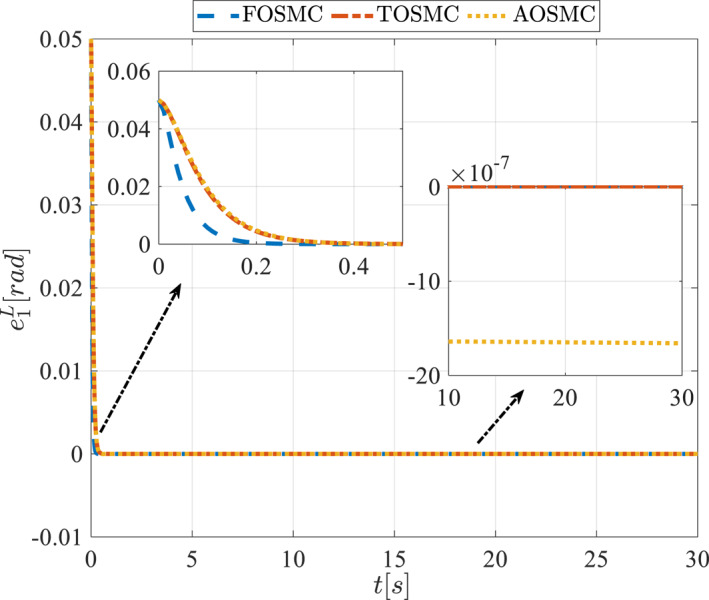
Tracking error of the first state of the pitching link in the first scenario.

**FIGURE 13:**
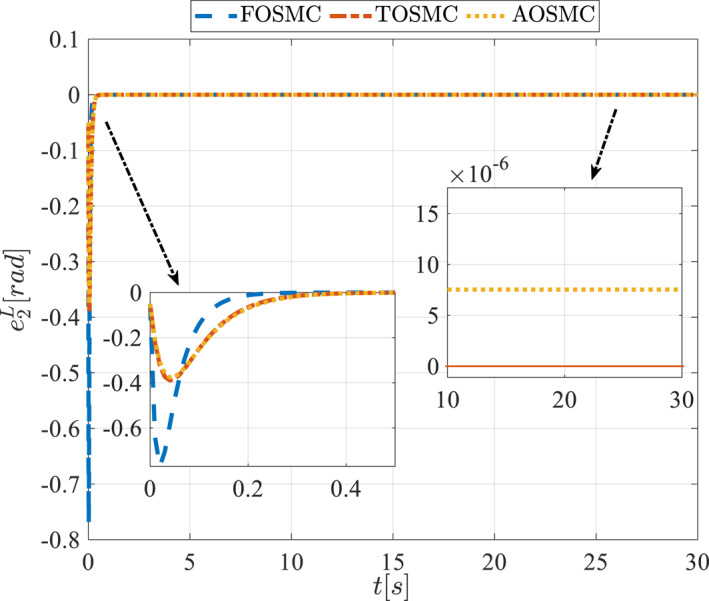
Tracking error of the second state of the pitching link in the first scenario.

**FIGURE 14:**
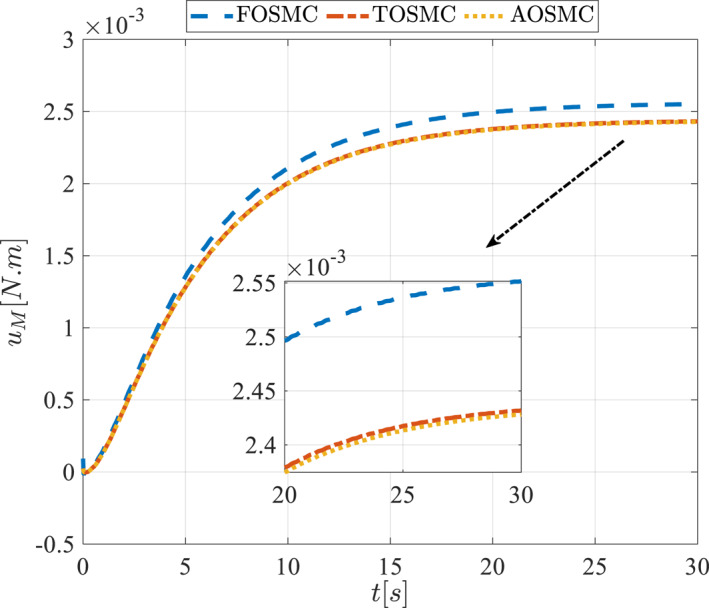
Control input for the driving pulley in the first scenario.

**FIGURE 15:**
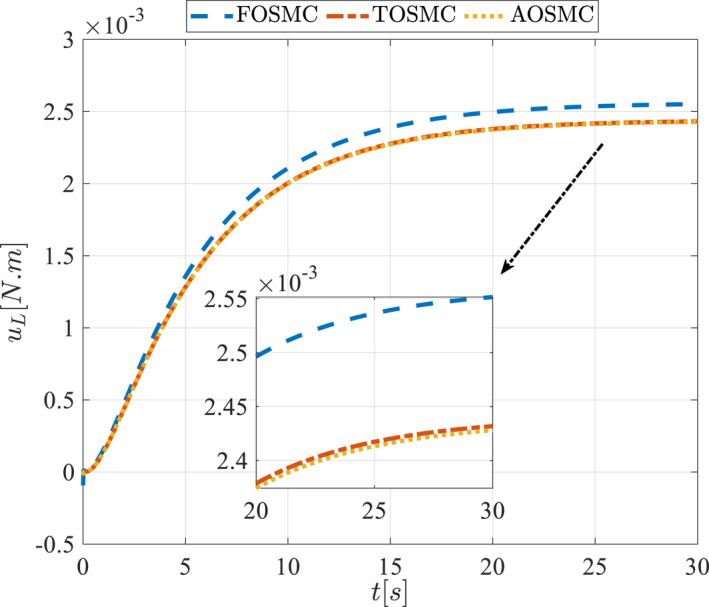
Control input for the pitching link in the first scenario.

**FIGURE 16:**
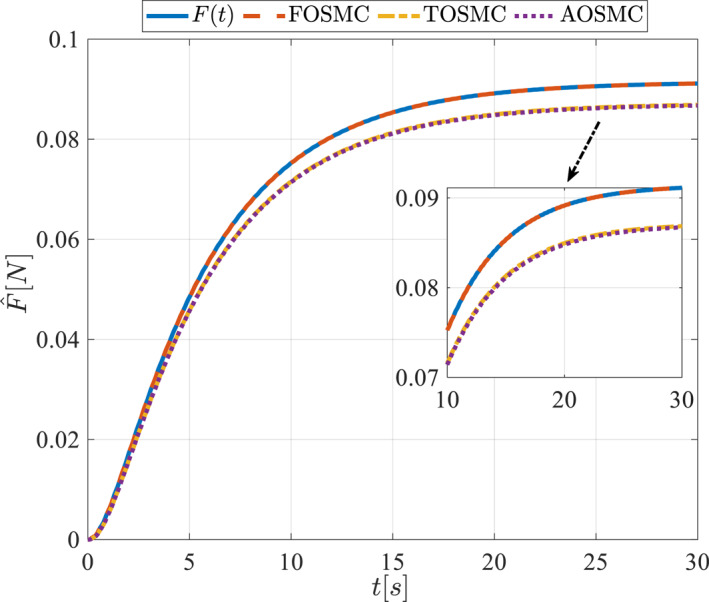
Estimation of the applied force in the first scenario.

**FIGURE 17:**
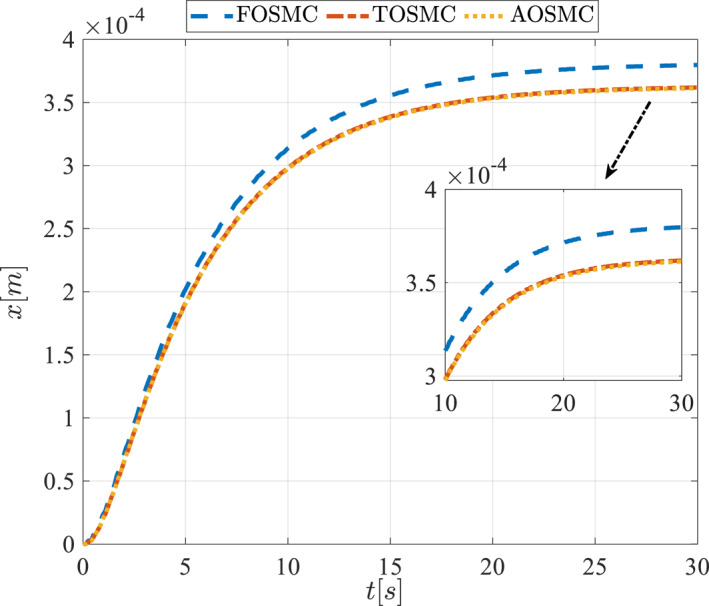
Eye displacement in the first scenario.

### 3.4 Second Scenario

**FIGURE 18:**
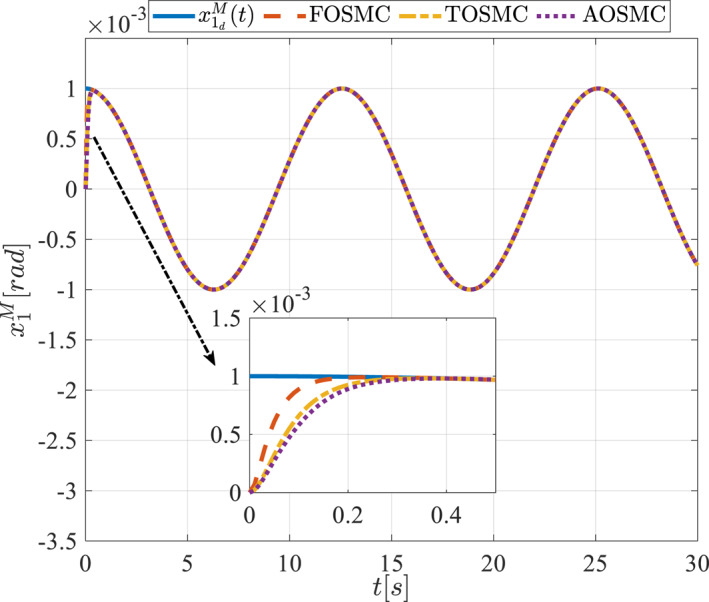
The first state of the driving pulley in the second scenario.

**FIGURE 19:**
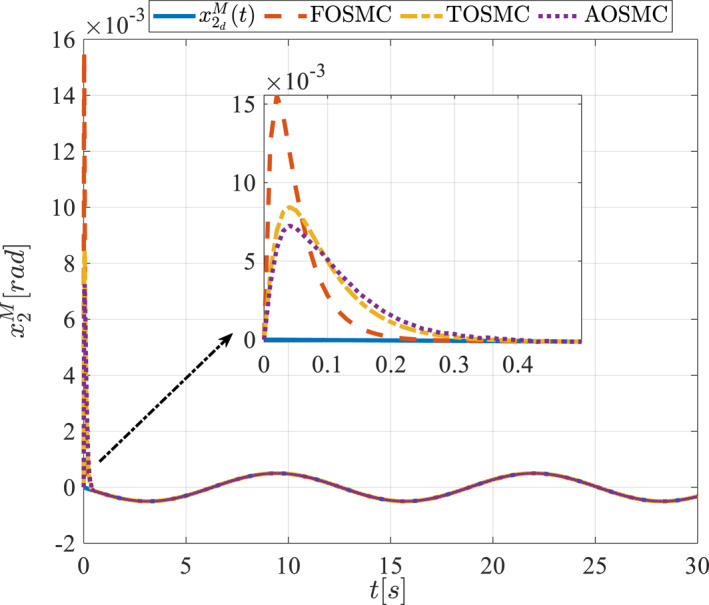
The second state of the driving pulley in the second scenario.

**FIGURE 20:**
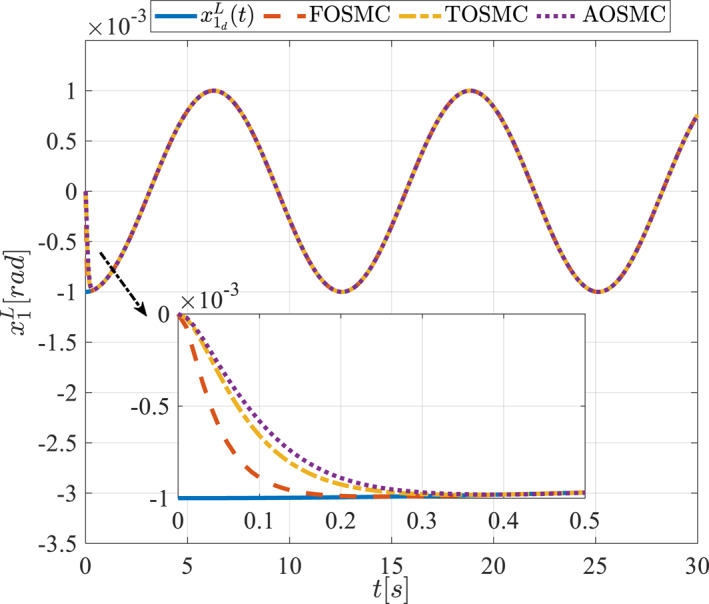
The first state of the pitching link in the second scenario.

**FIGURE 21:**
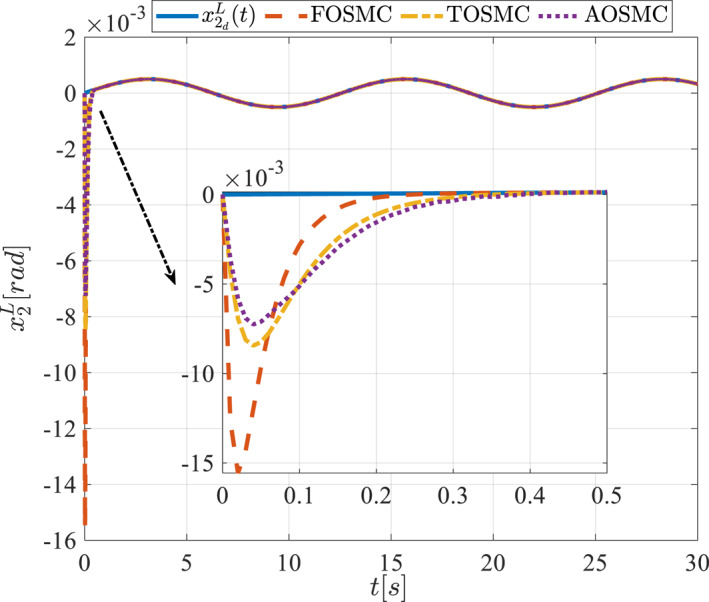
The second state of the pitching link in the second scenario.

**FIGURE 22:**
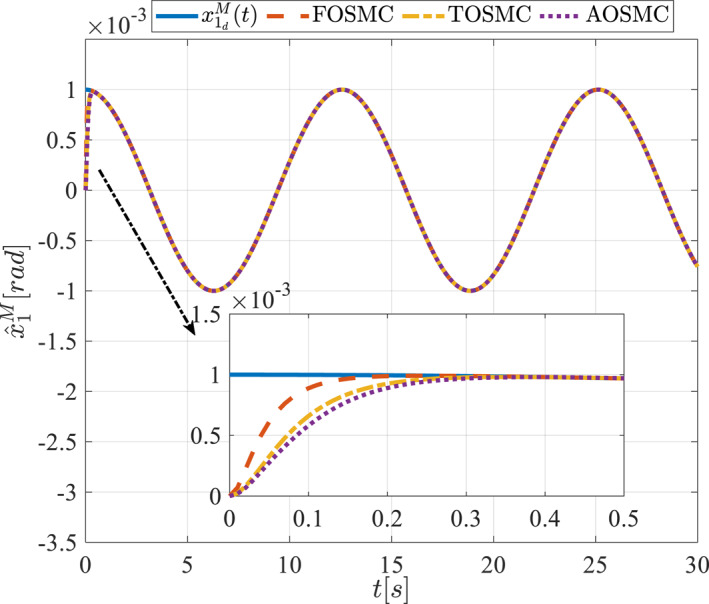
Estimation of the first state of the driving pulley in the second scenario.

**FIGURE 23:**
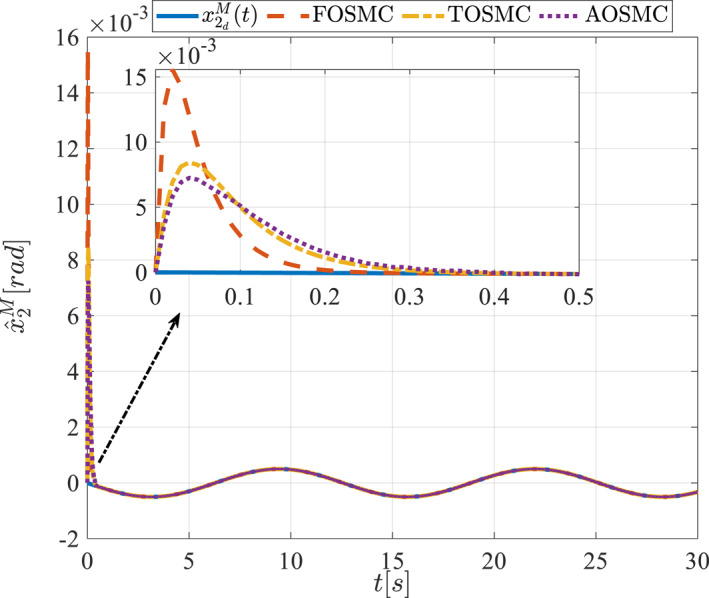
Estimation of the second state of the driving pulley in the second scenario.

**FIGURE 24:**
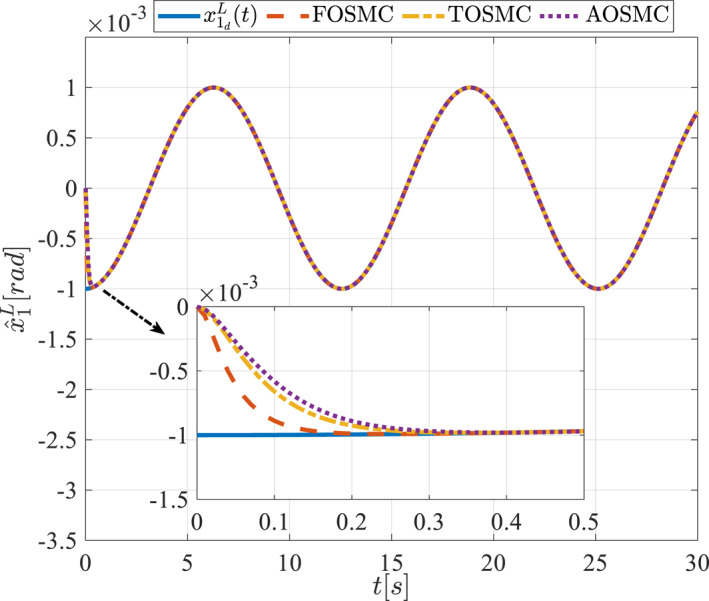
Estimation of the first state of the pitching link in the second scenario.

**FIGURE 25:**
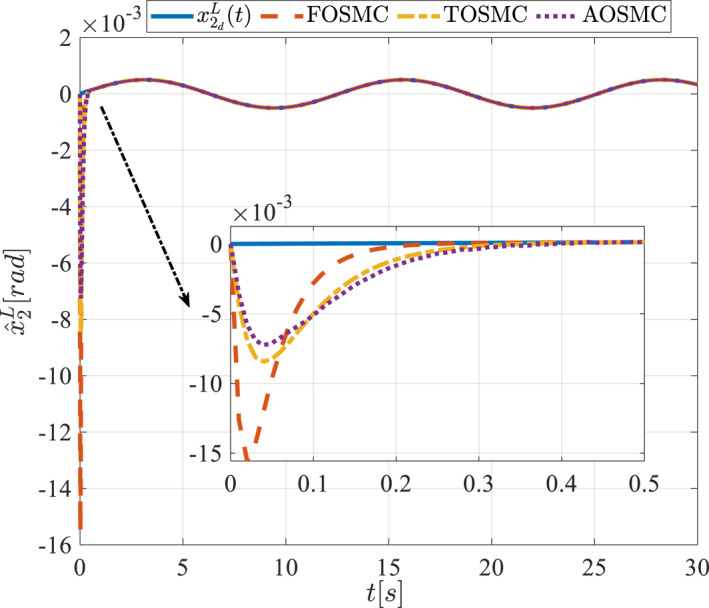
Estimation of the second state of the pitching link in the second scenario.

**FIGURE 26:**
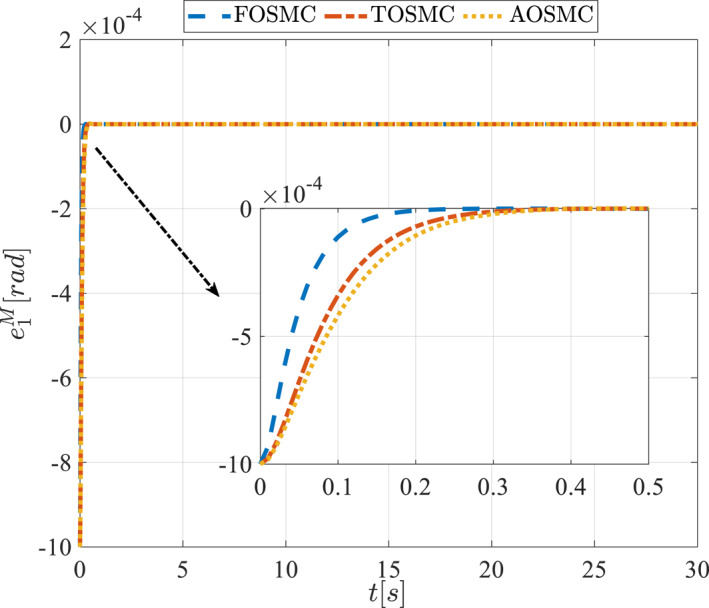
Tracking error of the first state of the driving pulley in the second scenario.

**FIGURE 27:**
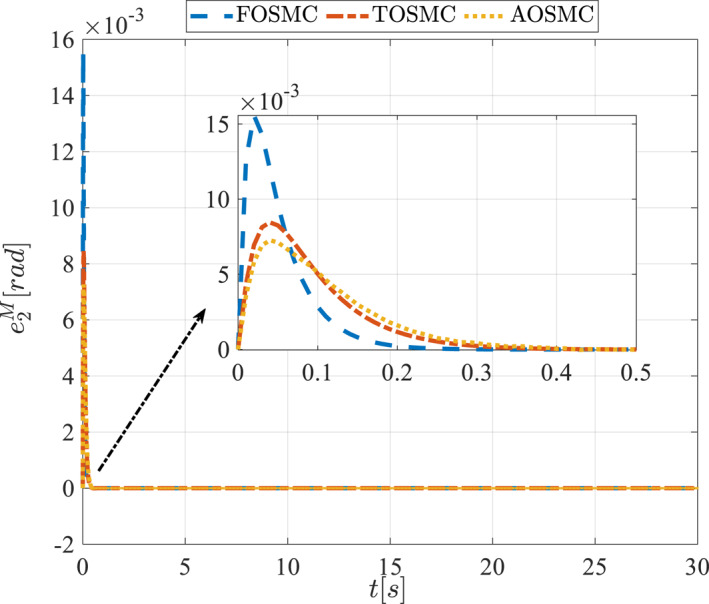
Tracking error of the second state of the driving pulley in the second scenario.

**FIGURE 28:**
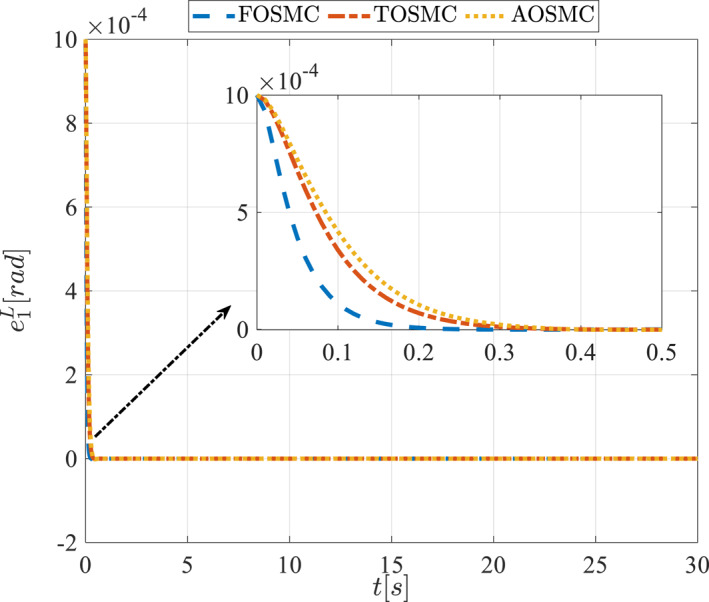
Tracking error of the first state of the pitching link in the second scenario.

**FIGURE 29:**
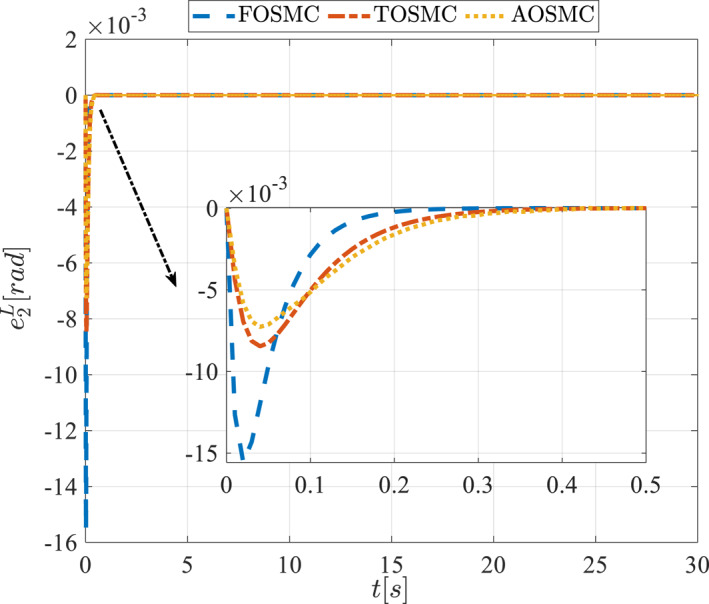
Tracking error of the second state of the pitching link in the second scenario.

**FIGURE 30:**
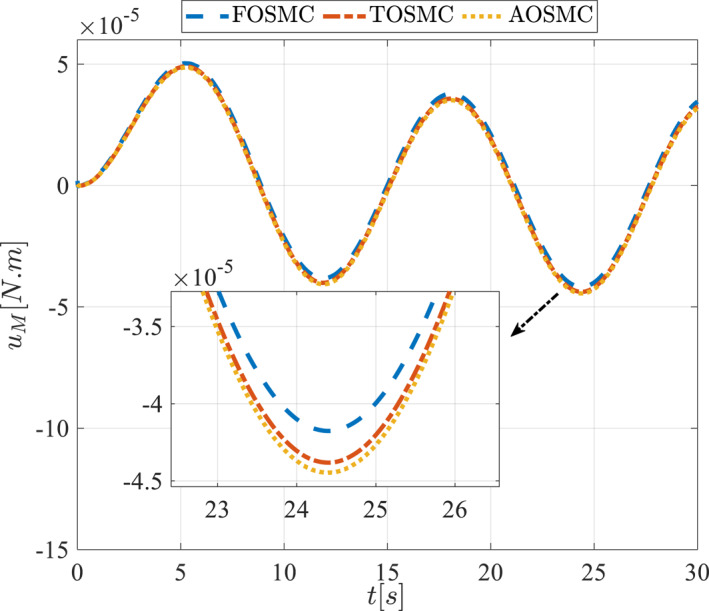
Control input for the driving pulley in the second scenario.

**FIGURE 31:**
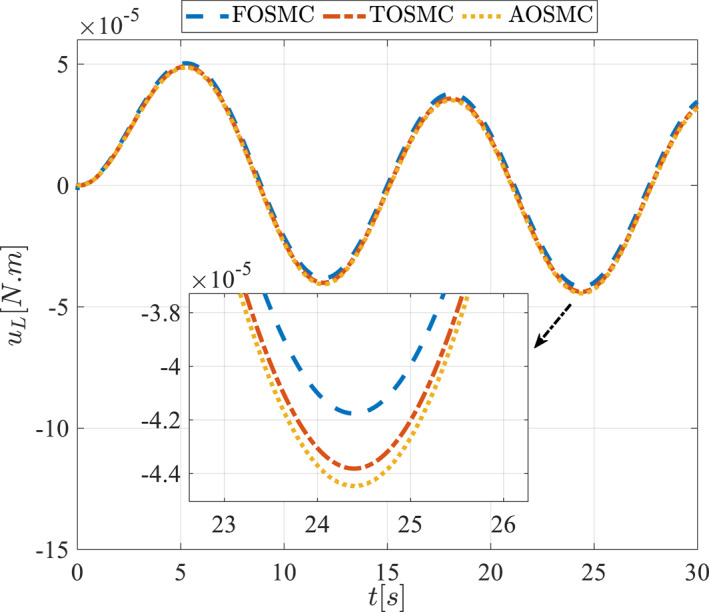
Control input for the pitching link in the second scenario.

**FIGURE 32:**
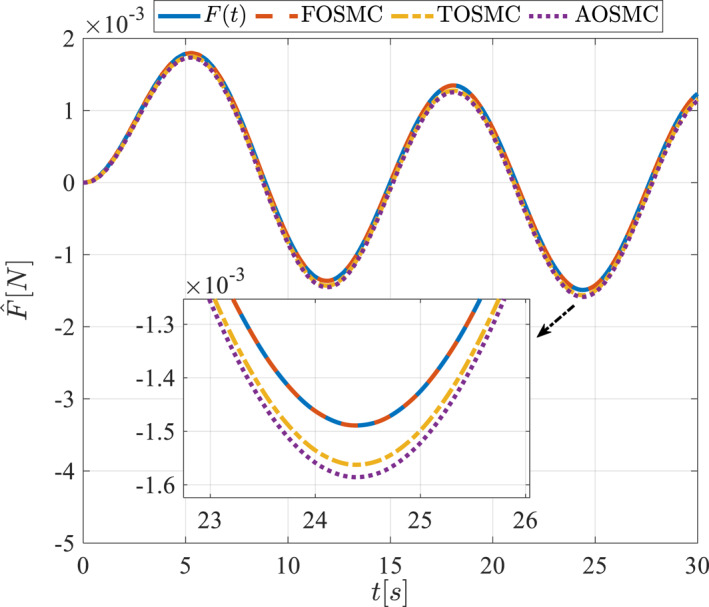
Estimation of the applied force in the second scenario.

**FIGURE 33:**
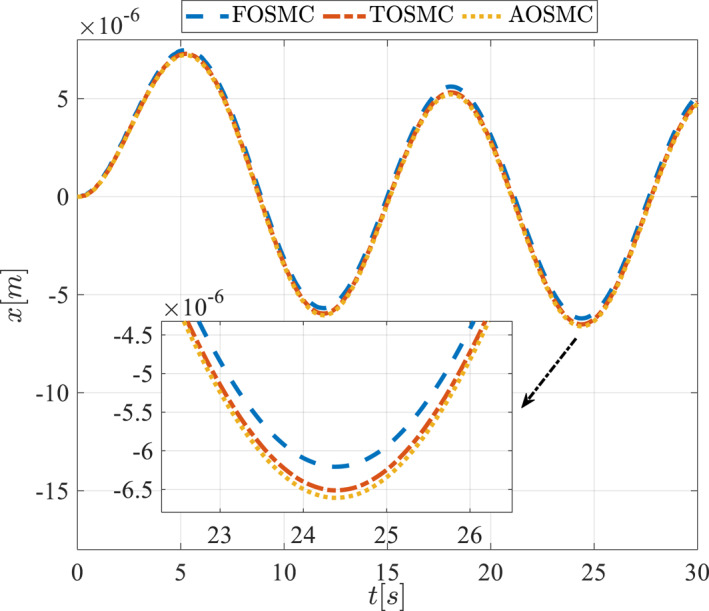
Eye displacement in the second scenario.

The second scenario was evaluated using Simulink/MATLAB software with the same control parameters as in the first scenario. This scenario has the same aims as the first scenario. The desired trajectories for this scenario are defined as $x_{1_{d}}^{M}=0.001\;\cos\left(0.5t\right),x_{2_{d}}^{M}=-0.0005\;\sin\left(0.5t\right),x_{1_{d}}^{L}=-x_{1_{d}}^{M},x_{2_{d}}^{L}=-x_{2_{d}}^{M}$. This is an example of a trajectory that could be a fragment of a movement of the gripper on the cornea. All methods were simulated under identical conditions. The simulation results of the second scenario are illustrated in Figure 18, 19, 20, 21, 22, 23, 24, 25, 26, 27, 28, 29, 30, 31, 32, 33.

## 4 Discussion

Figures 2, 3, 4, 5, Figures 18, 19, 20, 21 illustrate the states of the driving pulley and pitching link for the gripper's end‐effector along with their desired trajectories. The system states reach the desired trajectories rapidly and smoothly. The control parameters were chosen to achieve a settling time of less than 0.2 s. The same control parameters were applied to the comparison methods (where applicable). The zoomed‐in sections of the figures demonstrate that the proposed method provides a more accurate and faster response compared to the other two methods.

Figures 6, 7, 8, 9, Figures 22, 23, 24, 25 depict the estimated states alongside the desired trajectories. Fast tracking by the observers is essential in this study as the observers estimate the system states and relay them to the controller. The controller then uses these estimates to control the system.

Figures 10, 11, 12, 13, Figures 26, 27, 28, 29 present the tracking errors. These errors show that the proposed control method delivers a highly accurate, and fast response across all joints. Additionally, the proposed method outperforms other methods in terms of performance across all joints with significantly lower settling times.

For a gripper in robotic eye surgery of the corneal surface, the control signal must be smooth, have a low amplitude, and be chatter‐free. As demonstrated in Figures 14, 15, 30, and 31, the designed controller fulfils all these requirements. These figures show the control signal curves for the motors, which are smooth and practical for real‐world applications. The curves also confirm that the proposed controller is chatter‐free.

The cornea can tolerate only a limited amount of force, which must also be applied smoothly and with a controlled finite amplitude to avoid injury during surgery. Figures 16 and 32 illustrate the applied force and its estimation, demonstrating appropriate control for such gripper movements on the cornea, as depicted in Figures 17 and 33.

## 5 Conclusion

This paper presents a novel approach for controlling a gripper surgical instrument designed for a specific type of robotic eye surgery while estimating the applied force on the human cornea. The displacement of the cornea was calculated using a dynamic model of the human eye. The proposed method employed a fixed‐time stabilisation approach and was compared with two alternative techniques: finite‐time and asymptotic stabilisation methods. The stability of the proposed controller was proved using the Lyapunov theory. Simulations were conducted under two distinct scenarios, demonstrating that the proposed method delivers fast and accurate responses. The applied force was estimated in real‐time and incorporated into the control law as an online parameter. The proposed method relied on the gripper's estimated states to ensure safety during robotic eye surgery. Two control approaches, finite‐time and asymptotic stabilisation methods, were selected for comparison. The results were discussed based on several criteria, including settling time, steady‐state error, potential chattering in the control law, and the accuracy of force estimation. Future work will consider more advanced models, including variations in corneal thickness and differences in the radii of curvature for the cornea and sclera. The lens, located within the eyeball and not connected to the cornea, is not expected to significantly affect the applied force on the latter.

## Author Contributions

Conceptualization, A.S.S.A., A.O., B.P.; Methodology, A.S.S.A.; Software, A.S.S.A.; Validation, A.S.S.A., A.O., B.P.; Formal analysis, A.S.S.A., A.O., B.P.; Investigation, A.S.S.A., A.O., B.P.; Data curation, A.S.S.A., A.O., B.P.; Writing–original draft preparation, A.S.S.A.; Writing–review and editing, A.S.S.A., A.O., B.P.; Supervision, A.O., B.P.; Project administration, A.S.S.A., A.O., B.P.

## Ethics Statement

The authors have nothing to report.

## Conflicts of Interest

The authors declare no conflicts of interest.

## Data Availability

Data sharing not applicable to this article as no datasets were generated or analysed during the current study.
